# Interactions among *Trypanosoma brucei* RAD51 paralogues in DNA repair and antigenic variation

**DOI:** 10.1111/j.1365-2958.2011.07703.x

**Published:** 2011-05-25

**Authors:** Rachel Dobson, Christopher Stockdale, Craig Lapsley, Jonathan Wilkes, Richard McCulloch

**Affiliations:** University of Glasgow, College of Medical Veterinary and Life Sciences, Institute of Infection, Immunity and Inflammation, The Wellcome Trust Centre for Molecular ParasitologySir Graeme Davis Building, 120 University Place, Glasgow G12 8TA, UK

## Abstract

Homologous recombination in *Trypanosoma brucei* is used for moving variant surface glycoprotein (VSG) genes into expression sites during immune evasion by antigenic variation. A major route for such VSG switching is gene conversion reactions in which RAD51, a universally conserved recombinase, catalyses homology-directed strand exchange. In any eukaryote, RAD51-directed strand exchange *in vivo* is mediated by further factors, including RAD51-related proteins termed Rad51 paralogues. These appear to be ubiquitously conserved, although their detailed roles in recombination remain unclear. In *T. brucei*, four putative RAD51 paralogue genes have been identified by sequence homology. Here we show that all four RAD51 paralogues act in DNA repair, recombination and RAD51 subnuclear dynamics, though not equivalently, while mutation of only one *RAD51* paralogue gene significantly impedes VSG switching. We also show that the *T. brucei* RAD51 paralogues interact, and that the complexes they form may explain the distinct phenotypes of the mutants as well as observed expression interdependency. Finally, we document the Rad51 paralogues that are encoded by a wide range of protists, demonstrating that the Rad51 paralogue repertoire in *T. brucei* is unusually large among microbial eukaryotes and that one member of the protein family corresponds with a key, conserved eukaryotic Rad51 paralogue.

## Introduction

Homologous recombination (HR) is of central importance in the maintenance and transmission of the genome. The key factor in this process, termed RAD51 in eukaryotes, RecA in bacteria and RadA in archaea, appears to be universally conserved (Story *et al*., 1993; Brendel *et al*., 1997; Lin *et al*., 2006; Haldenby *et al*., 2009). HR is one of a number of DNA repair pathways that can reverse genotoxic damage, and acts in particular on DNA double-strand breaks (DSBs) (San Filippo *et al*., 2008; Kass and Jasin, 2010) and the restart of blocked or collapsed replication forks (Petermann and Helleday, 2010). Despite the core functions of HR, some organisms have co-opted the process for rearrangements targeted to a defined locus or loci, such as in mating type gene switching in fungi (Fraser and Heitman, 2003; Klar, 2007). Several pathogenic microbes, including the bacteria *Neisseria gonorrhoeae* and *Borellia sp.* and the eukaryotes *Babesia bovis* and *Anaplasma marginale*, use HR for antigenic variation, the changing of surface antigens to evade host immunity (Deitsch *et al*., 2009; Morrison *et al*., 2009). African trypanosomes, including *Trypanosoma brucei*, are protistan parasites of mammals belonging to the eukaryotic order Kinetoplastida and are responsible for devastating disease in humans and livestock in sub-Saharan Africa. African trypanosomes proliferate extracellularly in the bloodstream and tissue fluids of mammals and undergo antigenic variation of their major surface antigen, the variant surface glycoprotein (VSG) (Barry and McCulloch, 2001; Horn and Barry, 2005; Taylor and Rudenko, 2006; Morrison *et al*., 2009).

The success of antigenic variation in African trypanosomes relies upon each cell expressing one antigenic form of VSG at a time, and switching this to an antigenically distinct VSG. Such VSG switching, which is not induced by the host immune response, allows some of the infecting population to evade elimination by antibody-mediated killing, prolonging the infection and enhancing transmission to a new mammal, via the tsetse fly. Singular VSG expression in the mammal is achieved because a *VSG* gene must be present in a bloodstream *VSG* expression site (BES) for transcription. However, multiple BESs are found in the genome, and therefore a switch in the expressed VSG can be enacted by inactivating the fully transcribed BES and activating full transcription from one of the silent BESs (Vanhamme *et al*., 2001; Borst, 2002; Pays, 2005). The mechanisms of, and trigger for, this transcriptional, or *in situ*, switching reaction are not yet understood, but it appears to be a co-ordinated process (Chaves *et al*., 1999; Ulbert *et al*., 2002) and some factors have been described whose mutation elevates transcriptional switching frequency (Landeira *et al*., 2009) or alters its dynamics (Figueiredo *et al*., 2008). In addition to the *VSG*s in the 20 or so BES, the *T. brucei* genome contains > 1600 *VSG* genes (Berriman *et al*., 2005; Marcello and Barry, 2007a). Most are concentrated in arrays in the subtelomeres of *T. brucei* megabase chromosomes (of which there are 11 diploid copies) (Melville *et al*., 1998), where ∼ 85% are pseudogenes. Large numbers of *VSG*s are also found in minichromosomes and intermediate chromosomes, chromosome classes specific to African trypanosomes among the Kinetoplastida (Ersfeld *et al*., 1999; Wickstead *et al*., 2004). Such *VSG*s are transcriptionally silent unless activated by recombination into the BESs. The most commonly observed recombination reactions in VSG switching are gene conversions where a silent *VSG* is copied and displaces the *VSG* residing in the BES (Hoeijmakers *et al*., 1980; Pays *et al*., 1981; Robinson *et al*., 1999). *VSG* gene conversion can encompass more sequence that the *VSG* ORF: copies of silent array *VSG*s normally extend to upstream *VSG*-associated 70 bp repeats (Liu *et al*., 1983), while much greater amounts of sequence can be copied when the silent *VSG* is present in a BES (Laurent *et al*., 1983; Pays *et al*., 1983; McCulloch *et al*., 1997; Kim and Cross, 2010); in some cases the entire transcription unit is duplicated (Hertz-Fowler *et al*., 2008). Downstream, gene conversion can terminate in the *VSG* ORF or 3′ UTR, but can encompass the telomere if the silent *VSG* is at the chromosome end (de Lange *et al*., 1983). Other recombination reactions also act in VSG switching. Cross-over, or reciprocal, recombination reactions where telomeric *VSG*s are exchanged between chromosomes are found infrequently (Pays *et al*., 1985; Rudenko *et al*., 1996). Segmental gene conversions in which novel *VSG*‘mosaic’ genes are constructed from silent *VSG* ORFs also occur (Longacre and Eisen, 1986; Roth *et al*., 1986; Kamper and Barbet, 1992). Although mosaic *VSG* switching has been considered rare, it predominates late in *T. brucei* infections (Marcello and Barry, 2007a) and allows the parasite to use the huge numbers of *VSG* pseudogenes to construct intact *VSG*s. This is likely to be a key contributing reaction for the function of antigenic variation in trypanosome survival (Barbet and Kamper, 1993; Marcello and Barry, 2007b; Morrison *et al*., 2009).

Genetic analysis has uncovered a number of factors that direct *T. brucei* VSG switching by recombination. Mutation of RAD51, the catalytic recombinase in eukaryotic HR, or BRCA2, a mediator of RAD51 nucleoprotein filament formation, impairs VSG switching frequency (McCulloch and Barry, 1999; Hartley and McCulloch, 2008). More recently, mutation of TOPO3α, an orthologue of the *Saccharomyces cerevisiae* Top3 component of the Sgs1-Top3-Rmi1 complex (Chu and Hickson, 2009), was shown to elevate VSG switching frequency (Kim and Cross, 2010). These data suggest VSG switching, at least of intact *VSG* genes, is largely driven by an HR reaction in which strand exchange is mediated by RAD51 and recombination intermediates that lead to cross-overs are suppressed. This reaction mechanism is consistent with the suggestion that VSG switching may be promoted by DSBs in the 70 bp repeats of the actively transcribed BES (Boothroyd *et al*., 2009). Nevertheless, a number of findings may indicate VSG switching is a specific adaptation of HR. The Mre11-Rad50-Xrs2/Nbs1 complex is critical in sensing and processing DSBs during eukaryotic HR (Rupnik *et al*., 2010). *T. brucei* MRE11 mutants are impaired in DNA damage repair and recombination (Robinson *et al*., 2002; Tan *et al*., 2002), but show no discernible defect in VSG switching (Robinson *et al*., 2002). Similarly, mismatch repair is an important regulator of HR efficiency in eukaryotes (Jiricny, 2006), including *T. brucei*, but does not detectably influence VSG switching (Bell and McCulloch, 2003; Bell *et al*., 2004). Other observations suggest that VSG switching may exploit more than one HR pathway: although mutation of RAD51 or BRCA2 impairs VSG switching, *VSG* gene conversions can still be detected (McCulloch and Barry, 1999; Hartley and McCulloch, 2008); and ablation of the putative VSG switch-initiating 70 bp repeats from the active BES does not detectably impair VSG switching (McCulloch *et al*., 1997; Boothroyd *et al*., 2009).

Rad51-directed strand exchange in eukaryotic HR is regulated by a number of factors, including Brca2, Rad52, Rad59 and Rad54 (San Filippo *et al*., 2008). Among such HR ‘mediators’, the functions of Rad51 paralogues (Thacker, 2005) are least well understood. Rad51 paralogues are found in all eukaryotes (Lin *et al*., 2006) and are Rad51-like proteins that display much greater sequence divergence from Rad51 than Dmc1, another widespread Rad51-like protein that acts in meiosis (San Filippo *et al*., 2008). Rad51 paralogues are not specific to eukaryotes, as RadA-related proteins are found in archaea (Haldenby *et al*., 2009) and bacteria encode Sms/RadA, a RecA-like protein implicated in HR (Beam *et al*., 2002). Two Rad51 paralogues, Rad55 and Rad57, are found in *S. cerevisiae* and have been shown to heterodimerize and interact with Rad51 (Hays *et al*., 1995; Johnson and Symington, 1995), mediating Rad51 nucleoprotein filament formation or stability (Sung, 1997; Fortin and Symington, 2002). Mutants of Rad55 or Rad57 are sensitive to DNA damage and display reduced HR (Hays *et al*., 1995; Sugawara *et al*., 1995). In vertebrates five Rad51 paralogues are found, XRCC2, XRCC3, Rad51B (Rad51L1), Rad51C (Rad51L2) and Rad51D (Rad51L3), forming at least two complexes (Schild *et al*., 2000; Masson *et al*., 2001a,b; Liu *et al*., 2002). Although Rad51 paralogue mutants are lethal during mouse embryogenesis (Shu *et al*., 1999; Deans *et al*., 2000; Pittman and Schimenti, 2000; Kuznetsov *et al*., 2009), mutations of each gene in cultured cells demonstrate roles in DNA damage repair (Liu *et al*., 1998; Lio *et al*., 2004; Daboussi *et al*., 2005), chromosome stability (Cui *et al*., 1999; Smiraldo *et al*., 2005), HR (Johnson *et al*., 1999; Pierce *et al*., 1999; Brenneman *et al*., 2000; Drexler *et al*., 2004; Hatanaka *et al*., 2005) and Rad51 localization in subnuclear foci after DNA damage (Bishop *et al*., 1998; Takata *et al*., 2000; O'Regan *et al*., 2001; Takata *et al*., 2001; Tarsounas *et al*., 2004a; van Veelen *et al*., 2005). Like *S. cerevisiae* Rad55-Rad57, *in vitro* assays suggest some vertebrate Rad51 paralogues mediate Rad51 HR (Sigurdsson *et al*., 2001; Lio *et al*., 2003; Shim *et al*., 2004). *Arabidopsis thaliana* also encodes five Rad51 paralogues, with roles in DNA damage repair and meiosis (Bleuyard and White, 2004; Bleuyard *et al*., 2005; Osakabe *et al*., 2005), while four are found in *Drosophila melanogaster* (Ghabrial *et al*., 1998; Abdu *et al*., 2003; Radford and Sekelsky, 2004). The reasons that these multicellular organisms require an expanded repertoire of Rad51 paralogues is unclear, since *Caenorhabditis elegans* possesses a single such protein, Rfs1 (Ward *et al*., 2007). No work to date has looked beyond mammals and yeast to ask if Rad51 paralogues interact in functionally or structurally conserved complexes.

Previously, we described four *T. brucei* genes encoding putative RAD51 paralogues, as well as a DMC1 orthologue (Proudfoot and McCulloch, 2005; 2006;). Only two of the Rad51 paralogues (RAD51-3 and RAD51-5) were examined genetically and shown to have roles in *T. brucei* DNA repair and recombination. Surprisingly, only one of the two factors (RAD51-3) appeared to act in VSG switching (Proudfoot and McCulloch, 2005). To understand if each of the four genes indeed encode Rad51 paralogues, we describe here the consequences of mutating them individually. This confirms that all four genes contribute to DNA repair and recombination, although we show that their roles are not equivalent, suggesting distinct functions. In addition, we asked if the *T. brucei* RAD51 paralogues interact and if the complexes they form are comparable with those described in mammals and yeast. Finally, we examined the Rad51 paralogues that can be identified in the completed genome sequences of a wide range of parasitic and non-parasitic protists, microbial organisms that contribute much of the diversity of the eukaryotic kingdom, but where analysis of HR is limited (Bhattacharyya *et al*., 2005; Lopez-Casamichana *et al*., 2008; Lopez-Camarillo *et al*., 2009). In so doing, we show that the repertoire of Rad51 paralogues in *T. brucei* and related kinetoplastids is uniquely large among protsists, matched only by *Naegleria gruberi*, and detail a key member of the Rad51 paralogue family.

## Results

### Kinetoplastids contain an unusually large repertoire of RAD51 paralogues

**Fig. 1:**
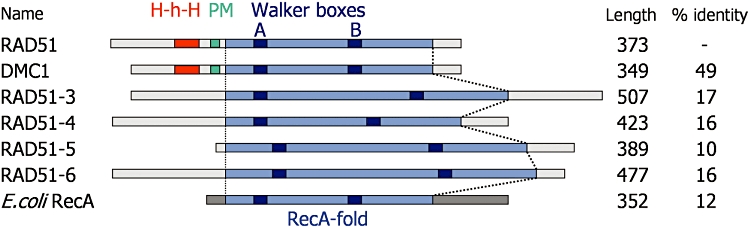
RAD51, DMC1 and RAD51 paralogues in *T. brucei*. Primary structures of *T. brucei* RAD51-related proteins relative to each other and to *Escherichia coli* RecA are shown; the proteins' lengths (amino acids) and sequence identity with *T. brucei* RAD51 are indicated. A conserved core (RecA-fold; light blue) is present in all proteins, containing Walker A and B motifs (dark blue). All the *T. brucei* proteins, except RAD51-5, contain N-terminal extensions that are not conserved with RecA; in this region RAD51 and DMC1 encode helix–hairpin–helix (H–h–H) and polymerization (PM) motifs not found in the RAD51 paralogues. A C-terminal extension in RecA is conserved with other bacteria RecA proteins, but is not conserved with the RAD51-related proteins.

In previous work, we described the RAD51 paralogues in *T. brucei* and the related kinetoplastid parasites *T. cruzi* and *Leishmania major* (Proudfoot and McCulloch, 2005); a summary of the domain organization and size of the *T. brucei* (Tb) proteins relative to TbRAD51, TbDMC1 and to *Escherichia coli* RecA is shown in Fig. 1, illustrating the divergence of the RAD51 paralogues from the catalytic recombinases. Since then, the genomes of a larger number of protists have been sequenced, including non-parasitic organisms (Fritz-Laylin *et al*., 2010). To ask what RAD51 paralogues are encoded by protists, we searched for RAD51 paralogues in a number of these genomes (Table 1). To do this, we used two strategies. First, we used blast searches with full-length TbRAD51 and TbRAD51-3 sequences, as well as with *S. cerevisiae* RAD51 and RAD57, and iterative searches within each genome using the RAD51-like proteins identified, manually searching for Walker A and B box motifs within a ‘RecA fold’, the conserved core of RAD51/RecA/RadA-like factors in eukaryotes, bacteria (Brendel *et al*., 1997) and archaea (Haldenby *et al*., 2009). Second, an alignment was extracted of the 40 most diverse sequences contributing to the Position Specific Scoring Matrix for the Conserved Domain Database (Marchler-Bauer *et al*., 2009) domain type cd01393 (RecA-like family including prokaryotic RecA, eukaryotic Rad51 and DCM1, and archeal RabA and RabB homologues). This alignment was then used to generate a Hidden Markov model using the hmmbuild program of HMMER2.3.2 (Eddy, 1998). Following calibration using the hmmcalibrate option this profile was used to search (hmmsearch, per-sequence Eval cutoff: ≤ 10) the UniprotKB peptide sequence database (current at January 2008). Virtually all the factors identified by the manual blast and annotation approach were found by this latter search (with Expectation values < 10^−30^), which additionally identified proteins clearly not Rad51/RecA-related, such as ABC multidrug resistance proteins and AAA+ ATPase factors (members of the P-loop containing nucleoside triphosphate hydrolase superfamily, Pfam CL0023) (Finn *et al*., 2006), all of which were discarded.

**Table 1:** A comparison of the presence (+), absence (−) or number of detectable RAD51, DMC1, RAD51 paralogue (Rad51par) and RecA proteins encoded by the genomes of a range of eukaryotic species, which are grouped into taxa and into five supergroups

Supergroup^a^	Taxon	Organism	RAD51	DMC1	RAD51par	RecA^b^
Excavata	Parabasalida	*T. vaginalis*	+	+	2	−
	Diplomonadida	*G. intestinalis*	−	2	−	−
	Euglenozoa	*T. brucei*	+	+	4	−
		*T. vivax*	+	+	4	−
		*T. congolense*	+	+	4	−
		*T. cruzi*	+	+	4	−
		*L. major*	+	+	3	−
		*L. braziliensis*	+	+	3	−
		*L. infantum*	+	+	3	−
	Heterolobosea	*N. gruberi*	+	+	3	−
Chromalveolata	Stramenopile	*T. pseudonana*	+	−	2	−
	Apicomplexa	*C. parvum*	+	+	2	−
		*T. annulata*	+	+	1	−
		*P. falciparum*	+	+	1	−
		*T. gondii*	+	+	1	−
	Ciliophora	*T. thermophila*	+	+	1	−
Plantae	Viridiplantae	*O. sativa*	+	+	5	3
		*A. thaliana*	+	+	5	3
Amoebozoa	Entamoebidae	*E. histolytica*	+	+	1	−
	Mycetozoa	*D. discoideum*	+	−	5	1
Opisthokonta	Microsporidia	*E. cuniculi*	+	−	1	−
	Fungi	*S. pombe*	+	+	4	−
		*S. cerevisiae*	+	+	2	−
		*U. maydis*	+	−	1	−
	Metazoa	*C. elegans*	+	−	1	−
		*D. melanogaster*	+	−	4	−
		*H. sapiens*	+	+	5	−
		*G. gallus*	+	+	5	−

a The supergroup Excavata is often split into two supergroups, one containing Euglenozoan, Heterolobosean and Jackobid (not shown) organisms, and the other containing Parabasalid, Diplomonad and Oxymonad (not shown) organisms.

b Where characterized, eukaryotic RecA proteins are targeted to the mitochondrion or chloroplast.

We consider that the combined output of these two bioinformatic approaches provides a comprehensive listing of canonical eukaryotic RAD51 paralogues in the genomes analysed (accession numbers or genome identifiers are provided in a supplementary file). None of the putative RAD51 paralogues showed evidence for helix–hairpin–helix domains, which were conserved in the N-termini of all the RAD51 and DMC1 orthologues (data not shown), illustrating their divergence from the recombinases. It is striking that the kinetoplastids possess among the largest repertoire of RAD51 paralogues among protists, and indeed among single-celled eukaryotes (Table 1). Each *Trypanosoma* species examined contains four syntenic *RAD51*-like genes that encode recognizable orthologues of RAD51-3, RAD51-4, RAD51-5 and RAD51-6 in *T. brucei*, while each *Leishmania* species encodes three (all lacking RAD51-5; see *Discussion*). Only in multicellular eukaryotes, or in the social amoeba *Dictyostelium discoideum*, were greater numbers of RAD51 paralogues identified. Three putative Rad51 paralogues were found in *N. gruberi*, the free-living protist whose genome has been sequenced that is most closely related to the Euglenozoa, which includes *T. brucei* (Fritz-Laylin *et al*., 2010). A further protein (ID 70092) was distantly related to Rad51, but appeared not to possess Walker A and B motifs, and did not therefore match our criteria for Rad51 paralogues.

### All four *T. brucei* RAD51 paralogues act in nuclear DNA repair and recombination

To date, the functions of only RAD51-3 and RAD51-5 have been examined among the RAD51 paralogues of any kinetoplastid (Proudfoot and McCulloch, 2005). To ask if RAD51-4 and RAD51-6 provide related functions, we made homozygous mutants of each gene in bloodstream stage *T. brucei*. To do this, and to allow comparison between all mutants, we followed the same gene disruption strategy adopted previously for *RAD51-3*, *RAD51-5* and *RAD51*, replacing the central core of the ORFs (including both Walker boxes) with antibiotic resistance cassettes (Fig. S1). *T. brucei* is diploid in the housekeeping core of the megabase chromosomes, and therefore two rounds of transformation were used for each gene, sequentially generating heterozygous (+/−) and homozygous (−/−) mutants that were selected via blasticidin and then puromycin resistance. Southern blotting and RT-PCR analysis showed that the gene disruptions had occurred as expected and that intact *RAD51-4* or *RAD51-6* genes and mRNAs were absent from the −/− clones (Fig. S1). To ensure reproducibility, two +/− mutants were generated independently for each gene, from which two independent −/− mutants were derived. In addition, we re-expressed RAD51-4 or RAD51-6 in the −/− mutants by targeting (via phleomycin selection) the intact ORF to the constitutively transcribed *tubulin* array [such re-expressers are designated −/−/+, and were verified by Southern blotting (data not shown) and RT-PCR; Fig. S1].

In common with *T. brucei rad51-3*−/− and *rad51-5*−/− mutants, *rad51-4*−/− and *rad51-6*−/− mutants were viable but displayed growth impairment relative to wild-type (wt) or +/− mutants. The population numbers of wt cells doubled *in vitro* on average in 6.69 (± 0.01) h, and +/− and −/−/+ cells grew at the same rate (data not shown). In contrast, *rad51-6*−/− mutants doubled in 9.52 (± 0.79) h, a growth impairment very similar to that measured in parallel for *rad51-3*−/− (9.80 ± 0.89 h) and *rad51*−/− cells (9.99 ± 1.0 h). *rad51-5*−/− and *rad51-4*−/− mutants showed population doubling times of 8.37 (± 0.31) h and 7.87 (± 0.73) h, which suggests somewhat less growth impairment than the other mutants, though still significantly different from wt (*P*-values each < 0.001; unpaired, two-tailed Student's *t*-test).

**Fig. 2:**
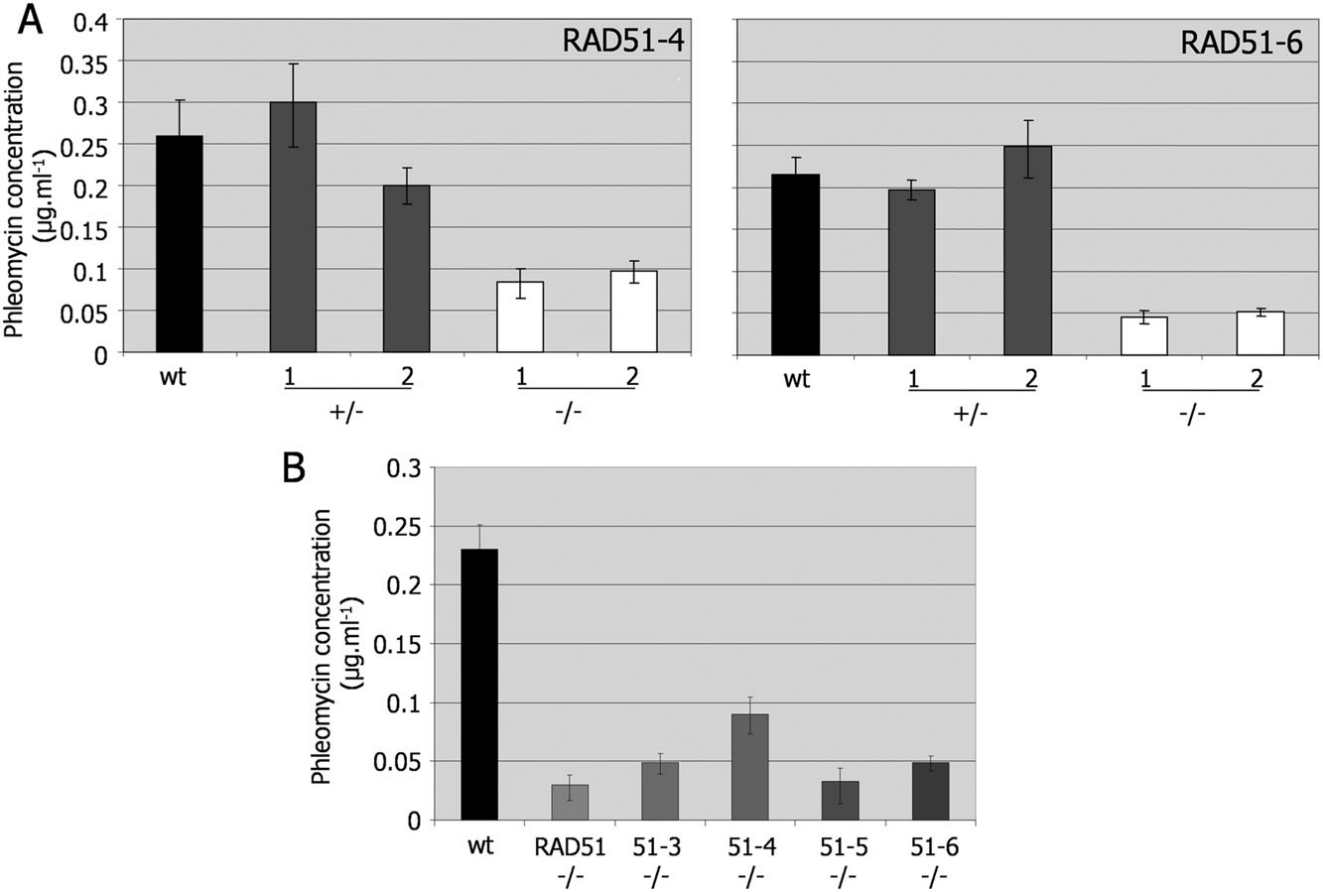
DNA damage sensitivity in *T. brucei* RAD51 and RAD51 paralogue mutants. A. The concentration of phleomycin that caused 50% growth inhibition (IC50) of wild-type (wt) cells is compared with two (1, 2) independent heterozygous (+/−) and homozygous (−/−) mutants of *RAD51-4* and *RAD51-6*. B. Comparison of the phleomycin IC50s of wt, *rad51*−/−, *rad51-3*−/−, *rad51-4*−/−, *rad51-5*−/− and *rad51-6*−/− cells. Values are averages from at least three experiments; bars indicate standard deviation.

To ask if RAD51-4 and RAD51-6 act in *T. brucei* DNA repair, we assayed the sensitivity of the mutants to exogenous DNA damage. First, we measured the survival of the cells in increasing concentrations of phleomycin, a glycopeptide antibiotic member of the bleomycin family that produces single-strand and double-strand breaks in DNA (Giloni *et al*., 1981). To do this, the IC50s of wt, +/− and −/− cells were determined over a wide range of phleomycin concentrations using Alamar Blue (Raz *et al*., 1997) as an indicator of metabolic function (Fig. 2A). In addition, we verified these findings using a clonal survival assay we have described previously (Conway *et al*., 2002a;Robinson *et al*., 2002; Proudfoot and McCulloch, 2005; 2006;), employing a more restricted range of phleomycin concentrations (Fig. S3). The *rad51-6*−/− mutants were approximately fourfold to sixfold more sensitive to phleomycin than the wt or *RAD51-6*+/− cells, consistent with an impairment in repair of such damage. *rad51-4*−/− mutants also displayed greater sensitivity to phleomycin damage than wt cells or their heterozygous antecedents, but to a lesser extant (approximately twofold to fourfold). To ask how these phenotypes of increased sensitivity compare with the other RAD51 paralogue mutants, and with RAD51 mutants, we measured the IC50s of all *T. brucei*−/− mutants (Fig. 2B), showing that each differed significantly from wt cells (*P*-values < 0.05 in all cases; unpaired, two-tailed Student's *t*-tests). These data also show that whereas *rad51-3*−/−, *rad51-5*−/− and *rad51-6*−/− mutants showed essentially equivalent increased sensitivity to phleomycin compared with each other and with the *rad51*−/− mutant, *rad51-4*−/− cells were less sensitive (*P*-values < 0.05, unpaired, two-tailed Student's *t*-tests). We next determined the sensitivity of the mutants to methyl methanesulphonate (MMS), an S_N_2 alkylating agent, using the same assays (Figs S2 and S3 for IC50s and clonal survival respectively). We found again that both *rad51-4*−/− and *rad51-6*−/− mutants were more sensitive to MMS than wt or +/− cells, and also demonstrated that re-expressing the protein was able to complement this phenotype. In addition, the *rad51-4*−/− mutants were the least sensitive of the mutants to MMS damage, although this was not as pronounced as seen for phleomycin. Taken together, these data indicate that all the RAD51 paralogues contribute to *T. brucei* DNA repair.

**Fig. 3:**
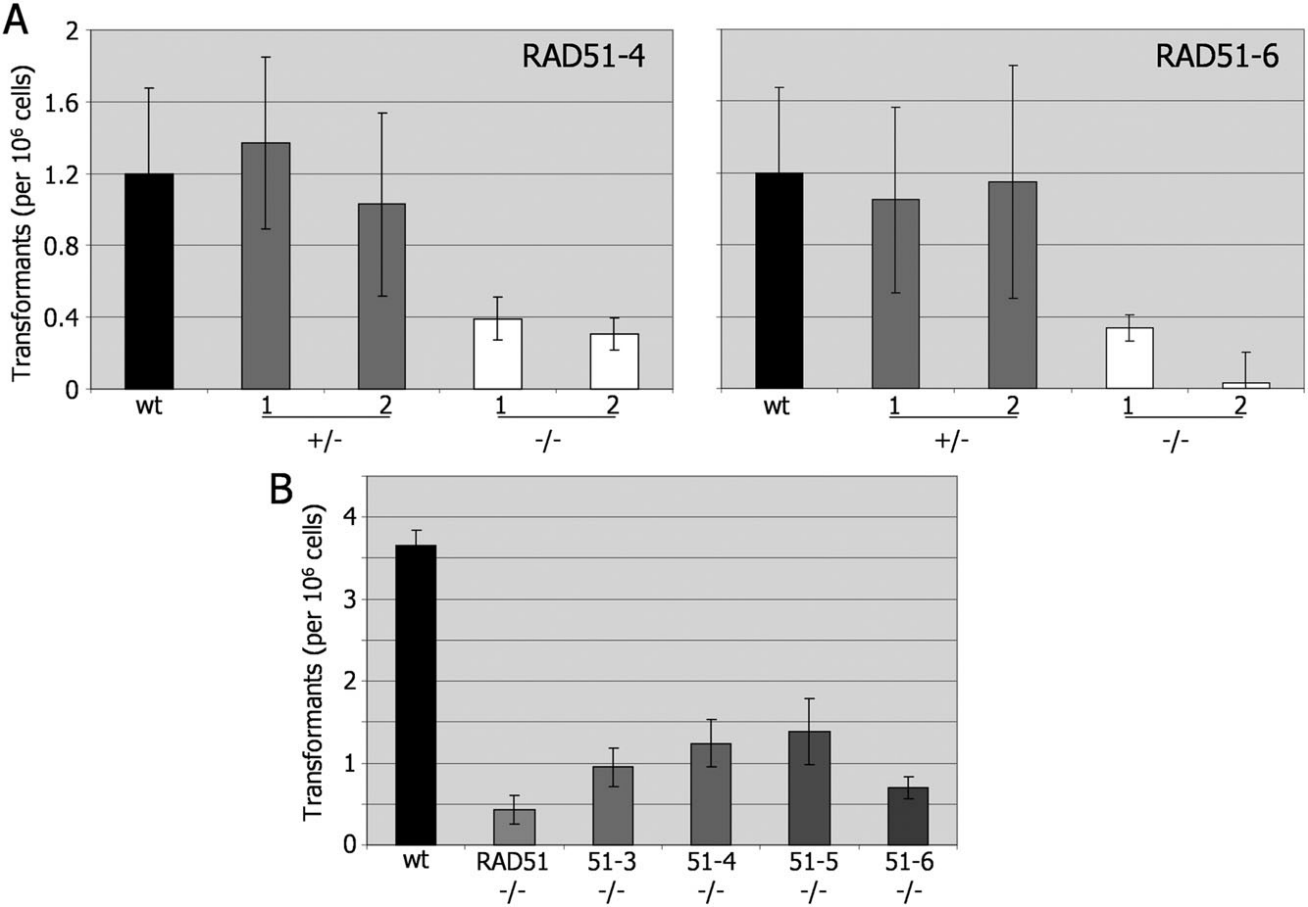
Homologous recombination in *T. brucei* RAD51 and RAD51 paralogue mutants. A. The number of transformants generated (per 10^6^ cells put on antibiotic selection) when the construct *tub-BLE-tub* was electroporated into wild-type (wt) *T. brucei*, or into two independent heterozygous (+/− 1, 2) or homozygous (−/− 1, 2) mutants of *RAD51-4* or *RAD51-6*, is shown. B. A comparison of the transformation frequency of wt, *rad51*−/−, *rad51-3*−/−, *rad51-4*−/−, *rad51-5*−/− and *rad51-6*−/− cells. Values are averages from at least three experiments; bars indicate standard deviation.

To ask if the above repair functions are due to the action of the RAD51 paralogues in *T. brucei* recombination, we measured the efficiency with which the mutants generate antibiotic-resistant transformants following electroporation with the construct *tub*-*BLE*-*tub*, which confers phleomycin resistance following HR-mediated integration into the *tubulin* locus. In a number of previous studies we have demonstrated the validity of this approach to measure *T. brucei* recombination frequency (Conway *et al*., 2002b; Robinson *et al*., 2002; Bell and McCulloch, 2003; Proudfoot and McCulloch, 2005; Barnes and McCulloch, 2007; Hartley and McCulloch, 2008), and that it is primarily a measure of HR efficiency as non-HR has never been observed in this organism (Burton *et al*., 2007; Glover *et al*., 2008). Figure 3A compares the transformation efficiency of the *rad51-4*−/− and *rad51-6*−/− mutants with +/− antecedents and with wt cells, and shows significant impairment in each homozygous mutant (*P*-values < 0.05 in all cases where the −/− cells were compared with wt or +/− cells by paired, two-tailed Student's *t*-tests). To compare the contribution to recombination of RAD51-4 and RAD51-6 with that of the other RAD51 paralogues and with RAD51 itself, the transformation efficiency of each −/− mutant was compared with each other and with wt cells. Because the effectiveness of phleomycin selection depends on oxygen and metal ion concentrations (Claussen and Long, 1999), transformation rates can vary between experiments. For this reason, we compared the transformation of the mutants in a single experiment using cells grown and selected after transformation in a single batch of medium, and using single preparations of DNA construct and phleomycin stock (Fig. 3B). These data show that each RAD51 paralogue −/− mutant is impaired in transformation, reflecting a reduced capacity for HR. None of the mutants, with the possible exception of *rad51-6*−/−, was as compromised in transformation as *rad51*−/− cells, perhaps consistent with supporting roles in the recombination reaction.

### *T. brucei* RAD51 paralogues act non-equivalently in RAD51 foci formation

To ask if all the *T. brucei* RAD51 paralogues contribute to the function of RAD51 during DNA repair, we next examined the capacity of the mutants to support the relocalization of RAD51 to subnuclear foci following DNA damage. Previously, we have shown in *T. brucei* that RAD51 can be detected in discrete foci in the parasite cell's nucleus following phleomycin treatment (Proudfoot and McCulloch, 2005; Hartley and McCulloch, 2008) or the generation of a site-specific DSB by the I-SceI meganuclease (Glover *et al*., 2008). The formation of such foci appears to be a conserved response to various forms of genotoxic damage in eukaryotes and bacteria (Kidane and Graumann, 2005; Renzette *et al*., 2005), and the structures are thought to represent the concentration of a number of repair enzymes at sites of damage (Lisby and Rothstein, 2009). The formation or stabilization of RAD51 foci is, furthermore, dependent on a number of factors in eukaryotes, of which RAD51-3, RAD51-5 and BRCA2 have been shown to play a role in *T. brucei* after phleomycin treatment (Proudfoot and McCulloch, 2005; Hartley and McCulloch, 2008).

**Fig. 4:**
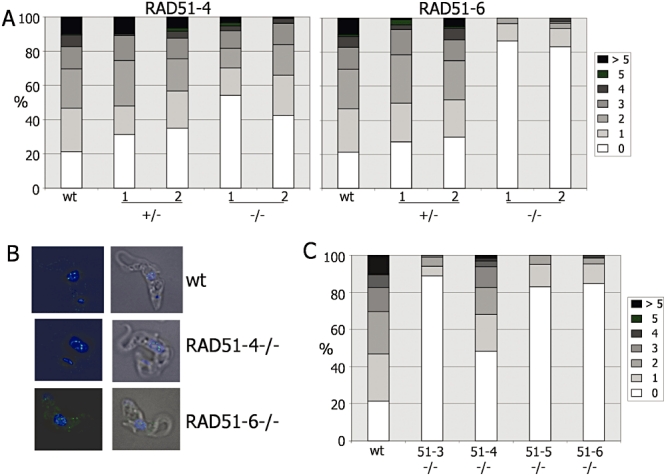
RAD51 subnuclear foci formation in *T. brucei* bloodstream form RAD51 paralogue mutants. A. Quantification of RAD51 foci formation in wild-type (wt) cells compared with two independent heterozygous (+/− 1, 2) or homozygous (−/− 1, 2) mutants of *RAD51-4* or *RAD51-6* after growth for 18 h in 1.0 µg ml^−1^ phleomycin; the number of subnuclear RAD51 foci in individual cells is shown as a percentage of the population (counting > 100 cells). B. Examples of RAD51 localization in wt, *rad51-4*−/−and *rad51-6*−*/*− heterozygous cells after 18 h growth in 1.0 µg ml^−1^ phleomycin. Cells were visualized by differential interference contrast (DIC), DNA was stained with DAPI, and RAD51 was visualized using polyclonal anti-RAD51 antiserum and SFX-conjugated goat-derived anti-rabbit secondary. Merged DAPI and RAD51 (left), and DAPI, RAD51 and DIC (right), images are shown. C. Quantification of RAD51 foci in wt, *rad51*−/−, *rad51-3*−/−, *rad51-4*−/−, *rad51-5*−/− and *rad51-6*−/− cells.

Figure 4A compares the number of RAD51 subnuclear foci detected by indirect immunofluorescence in *RAD51-4* and *RAD51-6* mutants after growth of the cells for 18 h in 1 µg ml^−1^ phleomycin. In the absence of phleomycin treatment, only a very small proportion (∼ 1–4%) of wt cells, or the +/− or −/− mutants, displayed RAD51 foci (data not shown) (Proudfoot and McCulloch, 2005; Hartley and McCulloch, 2008). In contrast, foci were seen in ∼ 80% of wt cells grown in phleomycin, with ∼ 50% displaying one to two foci and smaller numbers with ≥ 3 (an example of a wt cell with four foci is shown in Fig. 4B, and Fig. S4 shows examples of the range of foci formed). Although the protocol we have adopted here for RAD51 immunofluorescence is distinct from previous analyses (Proudfoot and McCulloch, 2005; Hartley and McCulloch, 2008), the DNA damage response in wt cells was very comparable. *RAD51-4*+/− and *RAD51-6*+/− cells also readily formed RAD51 foci, consistent with the lack of increased sensitivity to phleomycin-induced damage (Fig. 2). In contrast, *rad51-6*−/− cells were compromised in their ability to form RAD51 foci: in only 13–17% of the cells were RAD51 foci detectable, and this was primarily (10–11%) cells with a single focus (Fig. 4B). Because the *rad51-6*−/− mutants are more sensitive to phleomycin damage than wt or *RAD51-6*+/− cells, we also treated the cells with 0.25 µg ml^−1^ phleomycin. In these conditions, fewer foci were detected in wt (68% displayed foci, and 43% had only one focus) and *rad51-6*−/− cells (4–5% displayed one to three foci), showing that the impairment in RAD51 focal accumulation reflects the absence of RAD51-6 rather than abrogation of the DNA damage response due to increased cell death.

Figure 4A–C show that *rad51-4*−/− mutants are also compromised in their ability to form RAD51 foci, but that this is less marked than in other RAD51 paralogue mutants. After 18 h growth in 1 µg ml^−1^ phleomycin ∼ 45–58% of *rad51-4*−/− cells contained RAD51 foci, fewer than in wt (∼ 80%) or in *RAD51-4*+/− cells (66–68%), but considerably more than observed for the *rad51-6*−/− mutants. To ensure that this was not simply an effect seen at this concentration of drug, we analysed RAD51 foci after growth for 18 h in 0.25, 0.5 and 2.0 µg ml^−1^ phleomycin. The proportion of *rad51-4*−/− cells that contained RAD51 foci increased in parallel with increasing phleomycin, but was consistently lower than in wt cells (data not shown). To compare the contribution of the four RAD51 paralogues to RAD51 foci formation, each homozygous mutant was grown in 1 µg ml^−1^ phleomycin for 18 h (Fig. 4C), using a single preparation of medium and drug to avoid variation (see above). This confirmed our previous report that *rad51-3*−/− and *rad51-5*−/− mutants are impaired in RAD51 foci formation (Proudfoot and McCulloch, 2005), and highlights that *rad51-4*−/− mutants have markedly the least impairment in this process. Indeed, it is only in *rad51-4*−/− mutants that clearly defined RAD51 foci could be seen (see Fig. 4B and Fig. S5 for examples); in all the other mutants (Fig. 4C and Figs S5 and S6) punctate staining was more pronounced throughout the cells and may have been incorrectly scored as foci if it overlapped the nucleus. Finally, western blot analysis showed that RAD51 was expressed in each *RAD51* paralogue −/− mutant (Fig. S7), demonstrating that impairment in RAD51 foci formation is not a consequence of loss of the recombinase.

### *T. brucei* RAD51 paralogues act non-equivalently in antigenic variation

**Fig. 5:**
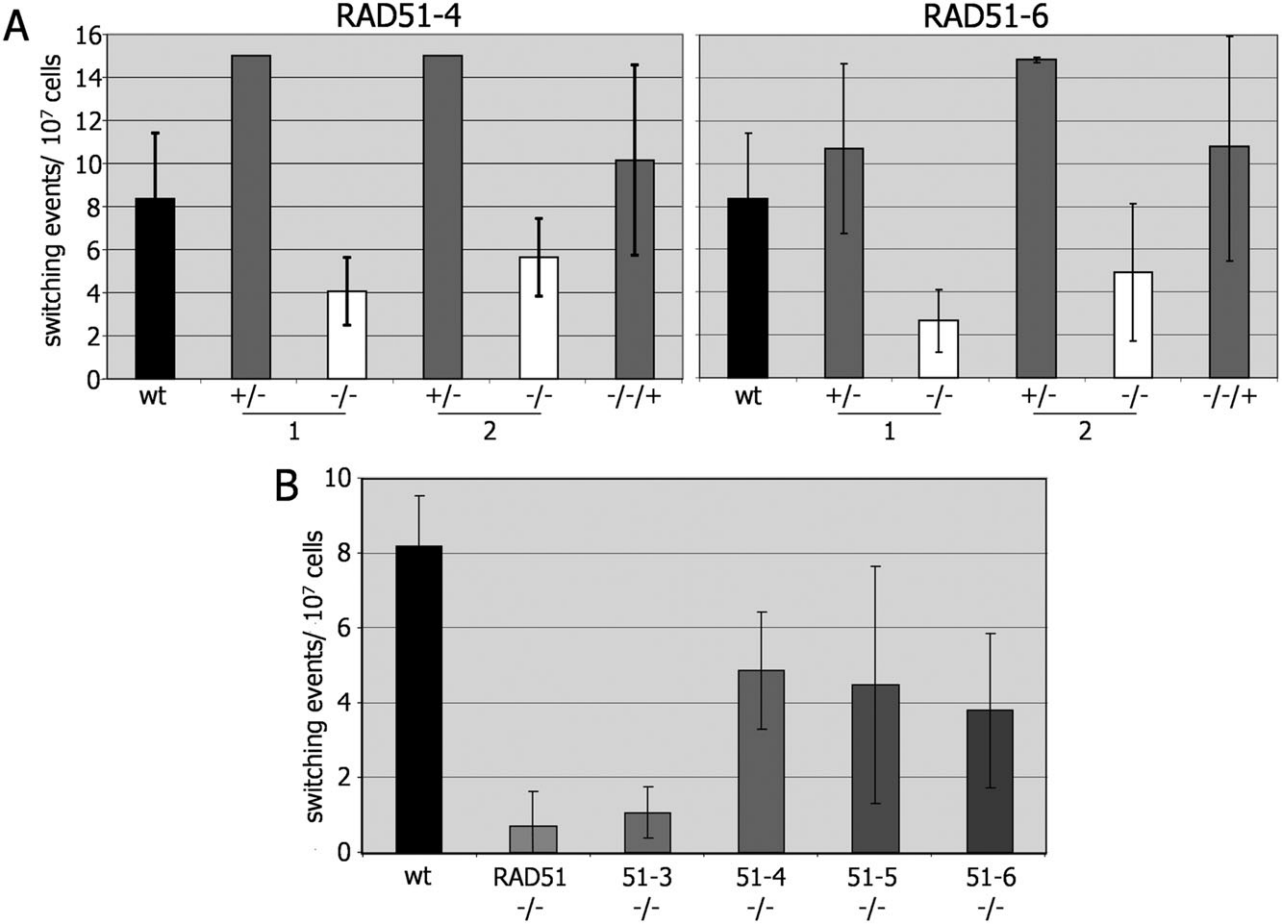
Effect of *RAD51* or *RAD51* paralogue mutation on the frequency of *T. brucei* VSG switching. A. Analysis of the effect if RAD51-4 or RAD51-6 mutation on VSG switching. The frequency with which VSG switch variants arose is shown in wild-type *T. brucei* cells (strain 3174.2; wt), two independent heterozygous (+/− 1, 2) or homozygous mutants (−/− 1, 2), and in −/− cells re-expressing the mutated gene (−/−/+). B. A comparison of VSG switching frequency in wt, *rad51*−/−, *rad51-3*−/−, *rad51-4*−/−, *rad51-5*−/− and *rad51-6*−/− cells. Values are the means of at least three independent experiments, and bars indicate standard deviation.

Given the roles of RAD51-4 and RAD51-6 in the repair of induced DNA damage and in HR, and their distinct contributions to RAD51 subnuclear focal accumulation after phleomycin treatment, we next asked if the factors act in *T. brucei* antigenic variation. As in previous studies, where we demonstrated a role for RAD51, RAD51-3 and BRCA2 in VSG switching (McCulloch and Barry, 1999; Proudfoot and McCulloch, 2005; Barnes and McCulloch, 2007; Hartley and McCulloch, 2008), RAD51-4 and RAD51-6 mutants were generated in the Lister 427-derived transgenic strain 3174, which allows VSG switching frequency and pathways to be measured from a number of switched variants that arise after a fixed number of generations growth *in vitro* (Rudenko *et al*., 1996; McCulloch *et al*., 1997) from a starting population that exclusively expresses a bloodstream expression site (BES1) containing *VSG 427-2* (also called *VSG221*, or MITat1.2) (Hertz-Fowler *et al*., 2008). The switch frequencies determined for the *RAD51-4* and *RAD51-6* mutants are shown in Fig. 5A, comparing the −/− mutants with wt and +/− cells, as well as with paralogue re-expresser cells (−/−/+) generated in one of the −/− mutants. In each case, the data indicate a trend of lower numbers of switched variants recovered in the −/− mutants. However, although the switch frequencies were significantly different when comparing each independent −/− line with its +/− antecedent (each *P*-value < 0.02; unpaired, two-tailed Student's *t*-tests), significant differences were not observed uniformly relative to the wt or −/−/+ cells.

To ask about the contribution of each RAD51 paralogue to antigenic variation, the switch frequencies of all the −/− mutants were compared with each other, and with *rad51*−/− mutants and wt cells. To do this, the switch frequencies of the two independent −/− mutants generated for each *RAD51* paralogue (*rad51-4*−/− and *rad51-6*−/− from this study; *rad51-3*−/− and *rad51-5*−/− from Proudfoot and McCulloch, 2005) were considered to be equivalent and switch frequencies averaged from all experiments performed (> 6 in each case). The switch frequency of the *rad51*−/− mutants was derived from three independent −/− mutants (nine experiments) (McCulloch and Barry, 1999), and the wt from 14 experiments (McCulloch and Barry, 1999; Proudfoot and McCulloch, 2005; Hartley and McCulloch, 2008). Taken together, these data show that *rad51*−/− and *rad51-3*−/− mutants show significantly impaired ability to undergo VSG switching, with average switch frequencies (0.7 × 10^−7^ and 1.1 × 10^−7^ switches per cell respectively) ∼ 12- and ∼ 8-fold reduced relative to wt (8.2 × 10^−7^ switches per cell). In contrast, *rad51-4*−/− and *rad51-6*−/− mutants were comparable with *rad51-5*−/− mutants in generating VSG switched variants approximately fourfold to fivefold more frequently than the *rad51*−/− or *rad51-3*−/− mutants and displaying, at best, only a minor impairment relative to wt cells. It seems likely therefore that RAD51-4, RAD51-6 and RAD51-5 either play no role in VSG switching, or that each individually provides a small contribution that the assay used here is too insensitive to detect. To ask further if RAD51-4 or RAD51-6 act in VSG switching, we assayed the relative contribution of recombination and *in situ* switching reactions, as described previously (McCulloch *et al*., 1997). No clear differences could be discerned in the *rad51-4*−/− or *rad51-6*−/− mutants relative to wt or +/− cells (Fig. S8). This result is perhaps not surprising, since in this *T. brucei* strain *rad51*−/−, *rad51-3*−/− and *brca2*−/− mutants, despite switching VSG less efficiently, display the same broad profiles of switching as wt cells (predominantly using either transcriptional switching or gene conversions from the silent BES, and using gene conversions limited to the region encompassing the *VSG* and upstream 70 bp repeats less frequently) (McCulloch *et al*., 1997; McCulloch and Barry, 1999; Proudfoot and McCulloch, 2005; Hartley and McCulloch, 2008). Nevertheless, this appears to rule out a major role for RAD51-4 or RAD51-6 in reaction pathway choice during VSG switching.

### Analysis of *T. brucei* RAD51 paralogue interactions

**Fig. 6:**
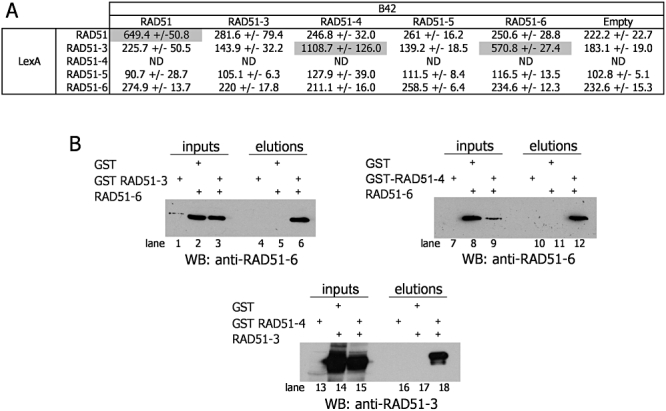
Interactions among *T. brucei* RAD51 paralogues and with RAD51. A. Yeast two-hybrid analysis of interactions. β-Galactosidase activity is shown for yeast cells carrying plasmids that express RAD51, RAD51-3, RAD51-4, RAD51-5 or RAD51-6 fused with a LexA DNA binding domain (LexA), in combination with plasmids expressing any of the proteins fused with a B42 transcriptional activation domain (B42); activity is also shown for control yeast cells carrying the LexA fusion-expressing plasmids and the B42 vector without a *T. brucei* gene (Empty). Values are the means of three independent experiments for two independent co-transformant yeast clones; deviation in activity around the mean is indicated (ND, not determined). B. Interactions between a GST–RAD51-3 fusion coexpressed in *E. coli* with native RAD51-6, or with a GST–RAD51-4 fusion coexpressed with native RAD51-3 or RAD51-6. Western blots (anti-RAD51 paralogue antiserum used is indicated by WB) are shown of the native proteins in cell extracts before purification (input), and after GST pull down of the GST fusion proteins from the cell extract (eluate) using glutathione beads. Controls are shown with GST fusion proteins expressed on their own, and with native RAD51 paralogues expressed with GST alone. Expression of the GST fusions, or of GST, by western analysis is not shown.

The above genetic analyses indicate distinct functions of the *T. brucei* RAD51 paralogues in some aspects of DNA repair and immune evasion. To ask if this is because each paralogue acts as an isolated protein, or whether they form a complex, or complexes, we next sought to examine interactions between the proteins. The ORF of each *RAD51* paralogue, and of *RAD51*, was cloned into the vectors pHybLex/Zeo and pYesTrp2 (Invitrogen), allowing yeast two-hybrid analysis by expressing the proteins as N-terminal fusions with the LexA DNA binding domain or with the B42 activating domain respectively. All pairwise combinations of pHybLex/Zeo-RAD51/paralogue and YesTrp2-RAD51/paralogue constructs were co-transformed into *S. cerevisiae* L40 cells and expression of each fusion protein confirmed by western analysis in two clonal co-transformants (data not shown). As a control, each of the pHybLex/Zeo-RAD51/paralogue vectors was also co-transformed with the pYesTrp2 vector alone. Interactions between the proteins were assayed via activation of a β-galactosidase reporter gene, by quantifying enzymatic activity in co-transformant lysates (Fig. 6A). The results were additionally verified by activation of a *HIS3* reporter gene, by testing for growth of the co-transformants on agar plates lacking histidine and containing 15 mM 3′ aminotriazole (data not shown). Only in those cells coexpressing LexA-RAD51 and B42-RAD51, or expressing LexA-RAD51-3 and B42-RAD51-4 or B42-RAD51-6, was there evidence for β-galactosidase activity above background (LexA fusion alone). This suggests that RAD51 is capable of self-interaction, but does not interact with the RAD51 paralogues in these conditions, and that RAD51-3 can interact with RAD51-4 and RAD51-6. The RAD51-3–RAD51-6 interaction appeared to be directional, as it was not detected in the co-transformants expressing LexA-RAD51-6 and B42-RAD51-3. No interactions between RAD51-5 and any other protein were detected. Finally, it is not clear from these data if RAD51-4 and RAD51-6 can interact, since the LexA–RAD51-4 fusion protein caused activation of β-galactosidase when expressed alone (∼ 3000–6000 units; 15- to 30-fold higher than the background level seen for all other proteins; data not shown). To attempt to dissect this, we expressed the N-terminal 151-amino-acid residues of RAD51-4 as a LexA fusion, as well as the C-terminal 330-amino-acid residues, based on previous work (Miller *et al*., 2004) that predicted distinct N- and C-terminal domains in human RAD51 paralogues that can modulate interactions. However, even the truncated RAD51-4 proteins caused self-activation of the reporter genes, precluding interaction analysis (data not shown).

To validate and extend the above yeast two-hybrid analysis, we next assayed for interactions between the RAD51 paralogues when expressed in *E. coli*. To facilitate this analysis, each of the *T. brucei* RAD51 paralogues was expressed in *E. coli* with 10 histidine residues fused to the C-terminus, allowing each protein to be purified and polyclonal antisera to be raised; western analysis with the recombinant proteins showed that each antiserum was specific to the cognate, purified RAD51 paralogue (Fig. S9). To analyse interactions, we coexpressed combinations of native (from the plasmid pRSF1b; Novagen) and N-terminal glutathione S-transferase (GST) fusions (from the plasmid pGEX-4T3; GE life sciences) of the RAD51 paralogues. Glutathione beads were used to recover the GST fusion proteins from *E. coli* extracts, and western analysis with the RAD51 paralogue antiserum was then used to determine if a coexpressed, native RAD51 paralogue was also recovered, indicating stable interaction. As controls, the native RAD51 paralogues were coexpressed with GST alone and subjected to the same purification, and GST–RAD51 paralogue fusions were expressed on their own and recovered in the same way (to control for antibody cross-reactivity). The results of this analysis are shown in Fig. 6B. This confirms the yeast two-hybrid data that showed RAD51-3–RAD51-4 and RAD51-3–RAD51-6 interaction (Fig. 6B, lanes 6 and 12 respectively). In addition, this approach revealed interaction between RAD51-4 and RAD51-6 (Fig. 6B, lane 18). Unfortunately, we were unable to assess whether or not RAD51-5 interacts with any of the other RAD51 paralogues, as GST–RAD51-5 fusions were insoluble and native RAD51-5 was not detected using the available antiserum when coexpressed with GST fusions of the other RAD51 paralogues. Given that it is possible that the RAD51 paralogues are able to bind DNA, we were concerned this may give artefactual co-purification during the centrifugation of the extracts. We therefore repeated the analysis and treated the extracts with DNAseI prior to GST purification, with the same results (data not shown), consistent with protein-mediated interactions.

**Fig. 7:**
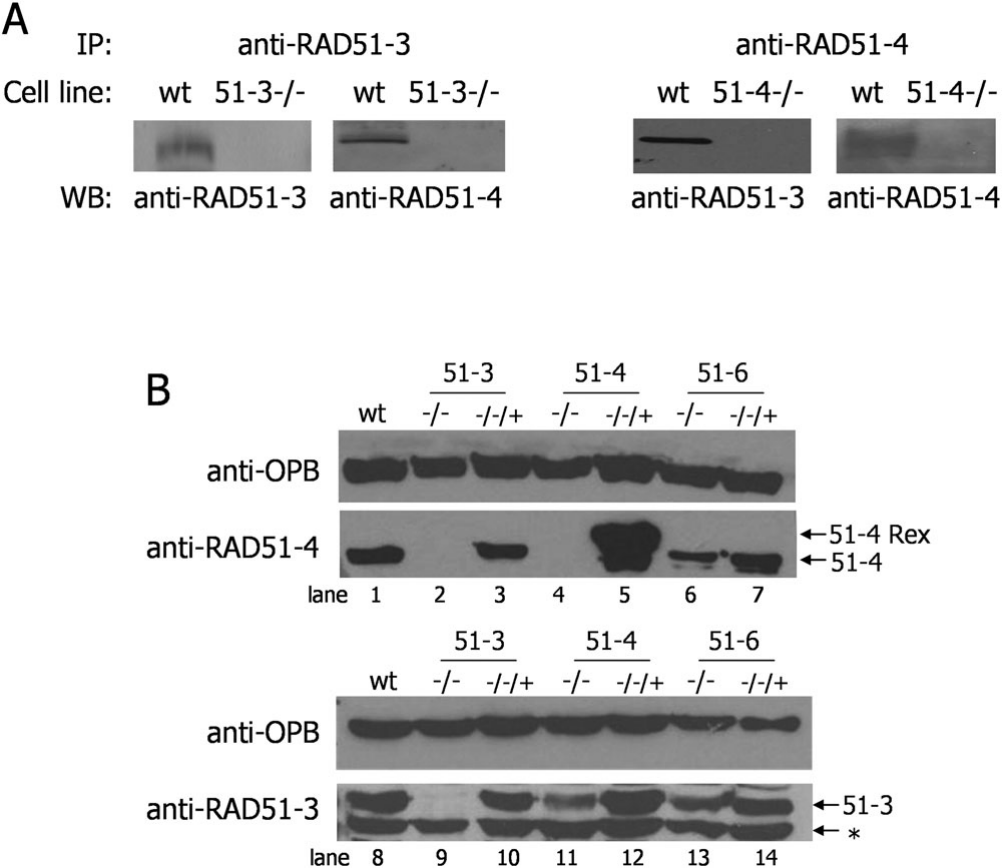
Interactions and expression interdependency of *T. brucei* RAD51 paralogues *in vivo*. A. RAD51-3 and RAD51-4 interact *in vivo*. Affinity-purified polyclonal antiserum against RAD51-3 or RAD51-4 was used to immunoprecipitate (IP) cognate protein from wild-type (wt) and *rad51-*3−/− or *rad51-4*−/− cells respectively. The presence or absence of RAD51-3 and RAD51-4 in all cases is shown by Western blots (WB) of the precipitated proteins, after SDS-PAGE, using anti-RAD51-3 and anti-RAD51-4 antiserum. B. Western blots are shown of whole cell extracts, after SDS-PAGE, from wt cells and from homozygous mutants (−/−) and re-expressers of RAD51-3, RAD51-4 and RAD51-6. Expression of RAD51-3 (51-3) or RAD51-4 (51-4) was assayed using affinity-purified polyclonal antiserum; a cross-reacting band is indicated in the anti-RAD51-3 blot (*), and a larger band seen only in the RAD51-4 re-expresser cell probed with anti-RAD51-4 antiserum is indicated (51-4Rex). As a loading control, the blots were stripped and re-probed with anti-OPB antiserum (gift, J. Mottram).

Having found evidence for interactions between the *T. brucei* RAD51 paralogues in heterologous systems, we now used the antisera against the proteins to attempt to ask if they form complexes *in vivo*. Western analysis of wt and RAD51 paralogue −/− mutants with each antiserum showed that only RAD51-3 and RAD51-4 could be detected in whole cell *T. brucei* extracts, even after affinity purification of each antiserum using cognate RAD51 paralogue protein purified from *E. coli* as a GST fusion (Fig. S9). Nevertheless, we performed immunoprecipitation with each antisera from wt and −/−*T. brucei* cells. Figure 7A shows that when RAD51-3 was immunoprecipitated from wt, but not *rad51-3*−/− cells, both RAD51-3 and RAD51-4 can be detected in the precipitated material, consistent with interaction. This was confirmed by immunoprecipitation of RAD51-4, which precipitated RAD51-3 also. In both of these immunoprecipitations, RAD51-5 and RAD51-6 could not be detected with the available antisera (data not shown). Immunoprecipitation with antiserum against RAD51-5 or RAD51-6 did not detect the cognate protein, nor any of the other RAD51 paralogues, including RAD51-3 or RAD51-4, so we suspect that these antisera are inadequate for this experimental analysis. Finally, we probed for RAD51 (readily detected in cell extracts; Fig. S7) in immunoprecipitates generated by the RAD51 paralogue antisera, and performed immunoprecipitation with anti-RAD51 antiserum and probed for the RAD51 paralogues in the immunoprecipitates, but found no evidence for interaction (data not shown).

### Expression levels of the RAD51 paralogues show interdependency

To probe potential interactions between the RAD51 paralogues further, we next asked if stable expression of the proteins is influenced by the presence or absence of the other factors. To do this, we concentrated on RAD51-3, RAD51-4 and RAD51-6, since we have evidence for interactions between the proteins *in vivo* and in heterologous systems. RAD51-3 and RAD51-4 protein levels were examined in Western blots of whole cell extracts from wt cells and compared with −/− mutants of each of the three genes and in re-expresser cell lines (−/−/+) (Fig. 7B). RAD51-4 was, as expected, undetectable in *rad51-4*−/− cells (lane 4). In addition, the protein was also absent in *rad51-3*−/− cells (lane 2). The same absence of RAD51-4 was not seen in *rad51-6*−/− cells (lane 6), although slightly lowered levels were apparent. Despite that fact that RAD51-4 abundance was strongly dependent on expression of RAD51-3, the opposite was not as clearly seen, despite considerable evidence that these proteins interact: RAD51-3 protein was detectable in *rad51-4*−/− cells (lane 11), though at lower levels than in wt or −/−/+*RAD51-4* cells. In addition, there was little evidence for alteration of RAD51-3 levels in *rad51-6*−/− cells (lane 13). Taken together, these data are consistent with the observations of interactions among these three *T. brucei* RAD51 paralogues. It suggests, furthermore, that the nature of the interaction between RAD51-3 and RAD51-4 renders RAD51-4 unstable or reduces gene expression in the absence of RAD51-3, while putative RAD51-3–RAD51-6 and RAD51-4–RAD51-6 interactions do not have as pronounced an effect.

## Discussion

In this study we have examined the functions of, and interactions among, RAD51 paralogues in the kinetoplastid parasite *T. brucei*, providing insight into the factors that contribute to DNA repair, recombination and antigenic variation in this important pathogen. In addition, this work provides an evolutionary perspective on the conservation of these recombination mediators (San Filippo *et al*., 2008), since functional analysis of recombination has been most extensive in fungi, vertebrates, plants and the nematode *C. elegans*, but less extensive in protists, which contribute a great deal of the evolutionary diversity of eukaryotes (Yoon *et al*., 2008). Within the RAD51 paralogues of *T. brucei* we show that one, RAD51-3, corresponds with the earliest emerging Rad51 paralogues in eukaryotes.

### Variability in RAD51 paralogue numbers in eukaryotes

Four RAD51 paralogues are found in African and American *Trypanosoma* species, as defined by identification of Walker A and B boxes within recognizable RecA-fold homology (Brendel *et al*., 1997). These proteins are present in addition to RAD51 and its meiotic cousin DMC1 (McCulloch and Barry, 1999; Proudfoot and McCulloch, 2006), and represent a larger number of RAD51 paralogues identified by sequence homology than in any other single-celled eukaryote thus far examined (excluding potentially highly diverged Rad51 paralogues recently described in *S. pombe*) (Martin *et al*., 2006). The work described here validates these bioinformatic predictions for *T. brucei*, showing that mutants of each predicted RAD51 paralogue are phenotypically impaired in repair of induced DNA damage, recombination of transformed DNA into the nucleus and relocalization of RAD51 to subnuclear foci following phleomycin treatment. What feature(s) of *Trypanosoma* biology may have selected for this expanded repertoire of nuclear repair factors is unclear. For instance, it appears not to reflect genome size, as *Trichomonas vaginalis* encodes only two Rad51 paralogues, despite having a genome (∼ 160 Mb) (Carlton *et al*., 2007) around fivefold larger than *T. brucei*, while *C. elegans* (∼ 100 Mb genome) has only one paralogue, Rfs-1 (Ward *et al*., 2007).

**Fig. 8:**
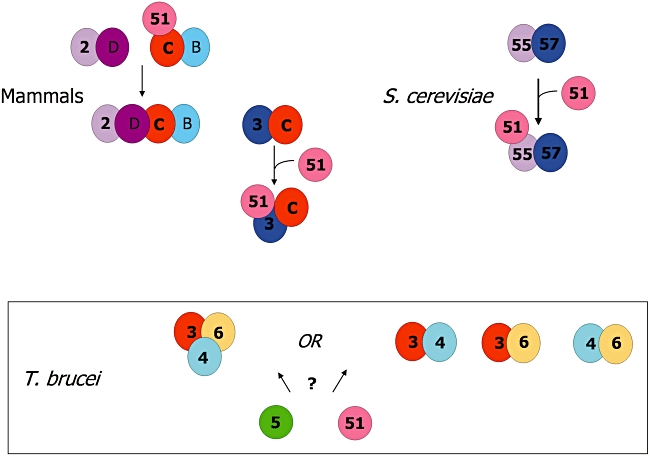
A comparison of RAD51 paralogue complexes, and their interaction with RAD51, in mammals, *S. cerevisiae* and *T. brucei*. The two principle Rad51 paralogue complexes found in mammals, XRCC2-Rad51D-Rad51C-Rad51B (2-D-C-B) and XRCC3-Rad51C (3-C), are shown as well as two further complexes (2-D, C-B). In *S. cerevisiae*, Rad55 (55) and Rad57 (57) are the only Rad51 paralogues, and form a stable complex. Experimentally determined interactions between the mammalian and yeast Rad51 paralogue complexes and Rad51 (51) are shown. In *T. brucei*, three Rad51 paralogues, RAD51-3 (3), RAD51-4 (4) and RAD51-6 (6), interact and experiments indicate either a stable trimer or distinct dimeric complexes; interactions with RAD51 or with a fourth Rad51 paralogue, RAD51-5 (5), have not been detected. Proteins are coloured to indicate potential orthology in the three organisms: Rad51 is orthologous in all (pink); Rad57 is considered orthologous with XRCC3 (dark blue) (Lin *et al*., 2006); and we propose that *T. brucei* RAD51-3 is orthologous with Rad51C (red).

In this study we have identified a broader range of eukaryotic RAD51 paralogues than has been documented to date (Table 1). To ask if these provide insight into the evolutionary origins of these proteins, we performed phylogenetic analyses that included bacterial RecA and archaeal RadA and RadB representatives (data not shown). In common with previous such analysis (Brendel *et al*., 1997; Lin *et al*., 2006; Haldenby *et al*., 2009), eukaryotic Rad51 and Dmc1 proteins (including all characterized protozoan Rad51 and Dmc1 orthologues; Table 1) grouped consistently with archaeal RadA, and bacterial RecA proteins grouped with organeller RecA-like proteins in eukaryotes, in each case suggesting common origins. The most complete analysis of eukaryotic Rad51 paralogue evolution to date is by Lin *et al*. (2006), who reported phylogenetic relationships primarily between vertebrates, plants and sea urchins, and found that the five Rad51 paralogues present in each grouped consistently, suggesting they were present in a common ancestor. Establishing the evolutionary relationships of the protistan Rad51 paralogues, including from *T. brucei*, was difficult as they did not consistently map to the well-supported groupings reported by Lin *et al*. This reflects the considerable sequence divergence of these proteins from each other and from the catalytic recombinases (Rad51, Dmc1, RadA and RecA). However, among protists that possess only one Rad51 paralogue (Table 1), these grouped most readily with Rad51C in some (e.g. *E. histolytica*) and XRCC3 in others (e.g. *P. falciparum* and *T. gondii*), both in phylogenetic analysis and as revealed by the ‘best’ hits in blast searches (data not shown). This seems consistent with the suggestion that these are the most ancient of the Rad51 paralogues (Lin *et al*., 2006). Perhaps surprisingly, given that mammalian Rad51C and XRCC3 interact (see below) (Masson *et al*., 2001a), in protists with two Rad51 paralogues, such as *T. vaginalis* and *C. parvum*, only one was identifiable as Rad51C-like or XRCC3-like, and the other was not easily assigned. *T. brucei*, *T. cruzi* and *Leishmania* sp., despite possessing more RAD51 paralogues, were consistent with this: in each, RAD51-3 most consistently grouped with Rad51C, and the other Rad51 paralogues had less certain orthology (Fig. 8; data not shown). Taken together, these data build upon the evolutionary picture of Lin *et al*. (Lin *et al*., 2006). Rad51C and/or XRCC3 appear to be the central, earliest emerging Rad51 paralogue(s), related to archaeal RadB, and in many protists these are the sole, extant orthologues. In other protists, further Rad51 paralogues are found. It is possible that gene duplication and diversification beyond the core Rad51C and/or XRCC3 Rad51 paralogue(s) occurred independently in different eukaryote lineages. It is alternatively possible that the overall smaller number of Rad51 paralogues found in most protists and fungi relative to animals and plants reflects gene loss from an initial repertoire of five Rad51 paralogues (Lin *et al*., 2006). Many sequenced protists are parasites and may therefore have undergone genome reduction similar to that seen in *Encephalitozoon cuniculi* (Gill and Fast, 2007), an intracellular microsporidian parasite related to fungi, though possessing only one RAD51 paralogue and lacking DMC1 (Table 1). Indeed, whole-genome comparisons between *N. gruberi*, a free-living protist that shares ancestry with kinetoplastids, suggest that parasitism has resulted in the loss of > 2000 conserved eukaryotic gene families in *T. brucei* (Fritz-Laylin *et al*., 2010). In this evolutionary context, the finding that *Trypanosoma* RAD51 paralogue numbers are potentially greater than in *N. gruberi* (Table 1) is striking. Irrespective of how the Rad51 paralogues evolved, the variability in their number and the extent of their sequence divergence appears to suggest considerable functional diversity, which we show is reflected in diversity in Rad51 paralogue interactions found in yeast, humans and *T. brucei* (see below). Moreover, it is possible that similar diversification has occurred during archaeal evolution, where variable numbers of RadA paralogues, including some distinct from RadB, have been described in a number of species (Haldenby *et al*., 2009).

### Interactions among *T. brucei* RAD51 paralogues

The work presented here indicates that at least three of the *T. brucei* RAD51 paralogues do not function in isolation. Instead, and in common with Rad51 paralogues from yeast and mammals, they are found as a complex or complexes. Yeast two-hybrid analysis demonstrated RAD51-3 interaction with both RAD51-4 and RAD51-6. These interactions were confirmed by *in vitro* GST pull down analysis, which additionally revealed interaction between RAD51-4 and RAD51-6. Finally, co-immunoprecipitation of RAD51-3 and RAD51-4 from *T. brucei* cell extracts confirmed interaction *in vivo*. Taken together, these data are compatible with these *T. brucei* RAD51 paralogues forming a single trimeric complex. Alternatively, interaction between the RAD51 paralogues may be more dynamic, with one or more dimeric sub-complexes in existence; Fig. 8 provides an extreme example, with three distinct dimeric sub-complexes, although we cannot discount there being one predominant dimer that associates with a third RAD51 paralogue in some circumstances. A number of observations suggest that a stable, trimeric complex is unlikely. First, we find that the contributions of RAD51-3, RAD51-4 and RAD51-6 to DNA repair are not equivalent, since *rad51-4*−/− mutants are significantly less sensitive to phleomycin-induced DNA damage than any other *T. brucei* RAD51 paralogue mutant. Second, and consistent with the above, *rad51-4*−/− mutants were markedly different from other *T. brucei* RAD51 paralogue mutants in that RAD51 foci numbers increased strongly following phleomycin-induced DNA damage, indicating a lesser role in either the formation or stabilization of these structures. Finally, we document variable levels of protein expression interdependency among the *T. brucei* RAD51 paralogues. Strikingly, RAD51-4 is not detectably expressed in a *rad51-3*−/− mutant, whereas RAD51-3 can be detected in a *rad51-4*−*/*− mutant. We are currently unable to assess RAD51-6 expression in these mutants, but find that in *rad51-6*−/− mutants RAD51-3 and RAD51-4 are both detectably expressed. These data are comparable with studies in human cells, where RNAi depletion of Rad51C resulted in similar depletion of XRCC3, but had less effect, if any, on the levels of Rad51D, Rad51B or RAD51, despite evidence that they also interact with Rad51C (Lio *et al*., 2004). Such expression interdependency presumably reflects co-stabilization of proteins in a heterodimer. The fact that *T. brucei* RAD51-4 stability is dependent on the presence of RAD51-3, but not vice versa, suggests that RAD51-3 is the central component of *T. brucei* RAD51 paralogue interactions. This appears telling, as it mirrors the centrality of mammalian Rad51C in two different complexes (XRCC2-Rad51D-Rad51C-Rad51B and XRCC3-Rad51C; Fig. 8), and is compatible with the proposed evolutionary relationship between *T. brucei* RAD51-3 and mammalian and plant Rad51C (see above).

Further work will be required to determine the topology of interactions between *T. brucei* RAD51-3, RAD51-4 and RAD51-6. We also have not yet been able to determine if *T. brucei* RAD51 interacts with any, or all, of these factors. This is possible (Fig. 8), as Rad51 has been documented to interact with mammalian Rad51C in the Rad51C-Rad51B sub-complex (Schild *et al*., 2000; Masson *et al*., 2001b; Lio *et al*., 2003) and with XRCC3 in the Rad51C-XRCC3 complex (Liu *et al*., 2002), while *S. cerevisiae* Rad51 binds Rad55 in the Rad55-Rad57 complex (Hays *et al*., 1995; Johnson and Symington, 1995; Sung, 1997). Equally importantly, it is unclear whether or not RAD51-5 interacts with the putative *T. brucei* RAD51 paralogue complexes. Yeast two-hybrid analysis failed to reveal any interactions between this factor and the other *T. brucei* RAD51 paralogues, or with RAD51, while technical details hampered our attempts to assay such interactions *in vitro* or *in vivo*. RAD51-5 is the smallest, most diverged protein in the *T. brucei* RAD51 paralogue family (Fig. 1). Homology searches of *Leishmania* species revealed only three RAD51 paralogues, with RAD51-5 not identified (Table 1). However, synteny analysis identified a gene on *L. major* chromosome 33 that is positionally conserved with *T. brucei RAD51-5* (chromosome 10; Fig. S10). Though encoding a protein with only distant homology with *T. brucei* RAD51-5 (∼ 14% amino acid identity), this appears to have arisen from a RAD51 paralogue, retaining a Walker B motif but lacking a Walker A motif or wider RecA-fold homology (Fig. S11). This may suggest recent, *Leishmania*-specific neofunctionalization of RAD51-5, perhaps consistent with a distinct role for RAD51-5 in kinetoplastid repair and recombination, which may have ceased to be important in the biology of *Leishmania* parasites.

Comparing the interaction data for mammalian Rad51 paralogues with the data we present here, and with the stable heterodimer found in *S. cerevisiae*, fails to reveal an evolutionarily conserved pattern. In mammals two primary complexes are found, XRCC2-Rad51D-Rad51C-Rad51B and XRCC3-Rad51C, and in addition two sub-complexes of the former, XRCC2-Rad51D and Rad51C-Rad51B (Masson *et al*., 2001a,b; Liu *et al*., 2002; Miller *et al*., 2002; Wiese *et al*., 2002). It has been argued that *S. cerevisiae* Rad57 is orthologous with XRCC3, and Rad55 with XRCC2 (Lin *et al*., 2006). The fact that the yeast proteins form a stable heterodimer (Sung, 1997) is then surprising, as no evidence has found direct interaction between XRCC2-XRCC3 in mammals, and they are not common to a complex (Fig. 8). The work here represents the only analysis of Rad51 paralogue interactions to date beyond yeast and mammals. We have argued that *T. brucei* RAD51-3 is Rad51C-like, but cannot confidently predict the orthology of RAD51-4, RAD51-5 or RAD51-6. We suggest that this reflects flexibility in Rad51 paralogue interactions in the eukarya. Although Rad51C or XRCC3-like factors appear central to Rad51 paralogue complexes when they form, the interacting factors are less conserved. Moreover, it appears that Rad51 paralogue complexes are needed only in some organisms, since a number of protists (Table 1) and examples of microsporidia, fungi and nematodes are found that appear to utilize only a single Rad51 paralogue (Table 1) (Kojic *et al*., 2006; Ward *et al*., 2007). This further underlines the flexibility in Rad51 paralogue evolution.

### Functions of *T. brucei* RAD51 paralogues?

The relatively broad phenotypic analysis of the *T. brucei* RAD51 paralogue mutants described here allows us only some insight into potential functions of these factors. Each contributes to the repair of phleomycin- and MMS-induced DNA damage, to the genomic recombination of transformed DNA constructs and to the relocalization of RAD51 into discrete foci following phleomycin damage. Although these contributions are not equivalent, since RAD51-4 appears less important in DNA repair and RAD51 foci dynamics, these data argue that each factor acts in RAD51-dependent HR. In any eukaryotic organism, the detailed functions of Rad51 paralogues are still being determined (San Filippo *et al*., 2008). In *S. cerevisiae*, mutation of Rad55 affects the recruitment of Rad51 to DNA damage (Sugawara *et al*., 2003), while the Rad55-Rad57 heterodimer promotes Rad51 strand exchange *in vitro* (Sung, 1997), consistent with mediating Rad51 function. In addition, Rad55 is phosphorylated in response to DNA damage, providing a checkpoint function that may be more important in stalled replication restart that in repair of an induced break (Herzberg *et al*., 2006). In mammals, each Rad51 paralogue complex contributes to Rad51-mediated repair, since mutations of any Rad51 paralogue results in increased DNA damage sensitivity (Takata *et al*., 2001) and attenuates Rad51 foci formation (van Veelen *et al*., 2005), similar to what we describe for *T. brucei*. Further work has begun to detail functions of the mammalian Rad51 paralogue complexes, consistent with mediating Rad51 function: Rad51B-Rad51C binds DNA and can promote Rad51 strand exchange (Sigurdsson *et al*., 2001); Rad51D-XRCC2 binds DNA (Kurumizaka *et al*., 2002); and both XRCC2-Rad51D-Rad51C-Rad51B and XRCC3-Rad51C bind to DNA junction structures in preference to intact DNA (Masson *et al*., 2001b; Yokoyama *et al*., 2004; Compton *et al*., 2010). However, increasing evidence also points to functions for Rad51 paralogues and their complexes beyond simply aiding Rad51 strand exchange. Mammalian Rad51C-XRCC3 has been associated with Holliday junction resolution (Liu *et al*., 2007), a late stage in recombination that may explain longer gene conversion tracts in mutants (Brenneman *et al*., 2002). Rad51C in mammals has been implicated in checkpoint signalling following DNA damage (Badie *et al*., 2009), in translocation of Rad51 to the nucleus (Gildemeister *et al*., 2009) and in mitosis (Renglin *et al*., 2007). Finally, mammalian Rad51D has been implicated in telomere maintenance (Tarsounas *et al*., 2004b), which may be a conserved function of the sole *C. elegans* Rad51 paralogue, RFS-1 (Yanowitz, 2008), which is considered to be Rad51D-like (Lin *et al*., 2006) and to play specific roles in recombination at replication blocks, rather than in general break repair (Ward *et al*., 2007). Given this, if the *T. brucei* RAD51 paralogues do indeed form a number of complexes, they may provide redundant functions in RAD51 strand exchange, or they may provide specific functions, perhaps in particular genomic contexts or in response to different DNA lesions.

In contrast to the equivalent contributions of *T. brucei* RAD51-3, RAD51-5 and RAD51-6 to the repair of phleomycin damage, the relative contributions of the proteins to the efficiency of VSG switching is distinct. Here, we find clear evidence for RAD51-3 acting to promote the process but, at best, only a minor contribution of RAD51-5 (Proudfoot and McCulloch, 2005), RAD51-6 and RAD51-4. Two explanations are possible. First, it is possible that the three RAD51 paralogue complexes we propose exist and each contributes to VSG switching. We have shown that *rad51-3*−*/*− mutants ablate both RAD51-3 and RAD51-4 expression, whereas RAD51-3 is expressed in *rad51-4*−*/*− and *rad51-6*−*/*− mutants, and RAD51-4 is expressed in *rad51-6*−*/*− cells. Thus, the greater effect of the *rad51-3*−*/*− mutation in VSG switching might arise because all complexes are functionally ablated, whereas RAD51-3–RAD51-6 and RAD51-3–RAD51-4 complexes remain in *rad51-4*−*/*− and *rad51-6*−*/*− cells respectively. If correct, this could either indicate redundancy in a specific step (such as RAD51-directed strand exchange), or that the complexes contribute to different recombination pathways that can fuel VSG switching. A complication in this scenario, and indeed in eukaryotic recombination in general, is BRCA2. This factor is also known to promote Rad51 strand exchange, most likely by co-ordinating the formation and disassembly of the recombinase nucleoprotein filaments (Lord and Ashworth, 2007; Jensen *et al*., 2010). In *T. brucei*, BRCA2 also promotes VSG switching, since *brca2*−*/*− mutants are defective to an extent comparable with *rad51*−*/*− and *rad51-3*−*/*− mutants (Hartley and McCulloch, 2008). To what extent BRCA2 function overlaps with, or even competes with, that of Rad51 paralogues is unknown. The second explanation is that RAD51-3 alone provides a VSG switching-specific recombination function. What this might be is unclear, but perhaps RAD51-3 acts on VSG switch-initiating lesions (Barry and McCulloch, 2009; Boothroyd *et al*., 2009), recombination intermediates (Kim and Cross, 2010) or links the process to cell cycle progression. Alternatively, recent work has shown that mammalian Rad51 paralogues can interact with other DNA repair mechanisms and cellular processes (Wilson *et al*., 2008; Rajesh *et al*., 2009), and perhaps this is the key to understanding *T. brucei* VSG switching.

## Experimental procedures

### *T. brucei* strains, growth, transformation and VSG switching

*Trypanosoma brucei* bloodstream cells of strain 3174, a derivative of MITat1.2a (McCulloch *et al*., 1997), were used throughout and grown at 37°C in HMI-9 medium (Hirumi and Hirumi, 1989). Cell concentration was determined with a haemocytometer (bright-line, Sigma). Electroporation of DNA constructs was conducted as described previously (Conway *et al*., 2002b; Bell and McCulloch, 2003). For both *RAD51-4* and *RAD51-6* mutants, transformants were selected with 0.5 µg ml^−1^ puromycin or 2.5 µg ml^−1^ blasticidin. Recombination was assayed by transformation with 5 µg of tub-BLE-tub (pRM450) digested with NotI and ApaI, and transformants selected with 2 µg ml^−1^ phleomycin, as described previously (Proudfoot and McCulloch, 2005). VSG switching frequency was determined as described previously (Proudfoot and McCulloch, 2005; Hartley and McCulloch, 2008). The type of VSG switching reactions used in the cells was determined by a combination of PCR and antibiotic resistance, as described previously (Proudfoot and McCulloch, 2005; Hartley and McCulloch, 2008), and no clear differences in pathway usage were observed in the mutants relative to wt (data not shown).

### Generation and analysis of *RAD51-4* and *RAD51-6* mutants

Constructs for gene disruption employed cassettes for puromycin *N*-acetyl transferase (puromycin) or blasticidin S deaminase (blasticidin) expression described previously (Hartley and McCulloch, 2008). These were flanked by 5′ and 3′ sequences derived from the *RAD51-4* or *RAD51-4* ORFs that had been PCR-amplified (primers available on request). For *RAD51-4*, the 5′ flank was 290 bp beginning at the predicted start codon; the 3′ flank was 402 bp and ended at the stop codon. For *RAD51-6*, the 5′ flank was also 290 bp, beginning at the start codon, while the 3′ flank was 354 bp, ending at the codon prior to the stop. All gene disruption constructs were digested with XbaI and XhoI before transformation. Re-expression constructs for *RAD51-4* and *RAD51-6* was generated by PCR-amplifying the genes' ORFs with Herculase polymerase and cloning them into EcoRV-digested construct pRM481. Drug-resistant transformants were analysed by probing Southern blots of restriction digested genomic DNA separated on 0.8% agarose gels, and using DNA fragments labelled using α-^32^P. For RT-PCR, total RNA was prepared from *T. brucei* using an RNeasy kit (Qiagen) and cDNA was synthesized with random hexamers and Superscript reverse transcriptase (Life Technologies). Primers to assay for gene expression are available on request.

### Assaying DNA damage sensitivity

Sensitivity of the cells to both MMS and phleomycin was assessed by the metabolic reduction of Alamar blue (Raz *et al*., 1997). Here, cells in mid-logarithmic growth were plated at a density of 1 × 10^5^ cells ml^−1^ in a 96-well dish (200 µl well^−1^) in HMI-9 medium containing doubling dilution concentrations of drug. After 48 h growth, 20 µl of Alamar blue (from a 12.5 mg ml^−1^ stock of resazurin, Sigma) was added to each well and the plates incubated in the dark for 24 h, when the fluorescence in each well was determined fluorometrically (PerkinElmer LS55) at 530 nm excitation wavelength and 590 nm emission wavelength. In addition, the sensitivity of the cell lines to the agents was analysed by a clonal survival assay that has been described before (Conway *et al*., 2002a; Robinson *et al*., 2002; Proudfoot and McCulloch, 2005; 2006;) (data not shown).

### RAD51 immunolocalization

RAD51 localization was performed by a modified technique to that described previously (Proudfoot and McCulloch, 2005; Hartley and McCulloch, 2008). Cells were grown to a density of approximately 1 × 10^6^ cells per millilitre, and pelleted at 600 *g* for 1 min. The supernatant was removed, and the cells were resuspended in 100 µl PBS (Sigma). 1 ml of 1% (v/v) formaldehyde/PBS was added and the solution inverted several times and then stored at 4°C for 1–24 h. The formaldehyde-fixed cells were then centrifuged at 600 *g* for 1 min at 4°C. The pellet was washed twice in 1 ml chilled PBS, followed by addition of 1% Triton X-100 (Sigma) to a final concentration of 0.1%. This was incubated at room temperature for 10 min, then glycine (Sigma) was added to a final concentration of 0.1 M and incubated at room temperature 10 min. The cells were pelleted and resuspended in 200 µl of PBS and spread evenly onto a glass slide (Menzel-glaser) and allowed to dry for 2–18 h. The slides were next washed with 1 × PBS for 5 min in Coplin jars and then blocked with TB buffer (0.1% Triton X-100, 0.1% BSA in PBS) for 15 min. This was removed and rabbit polyclonal anti-RAD51 antiserum, diluted 1:500 in TB buffer, was added and left for 55 min. The slides were then washed three times in PBS, 5 min each time, and secondary antiserum (goat anti-rabbit conjugated with SFX; fluorescein, succinimidyl ester, Molecular Probes), diluted in TB buffer, was added for 45 min in the dark. The slides were then washed as before and counter stained with two drops of VectorShield with 4,6-diamidino-2-phenylindole (DAPI; Vector Labs). A coverslip was then placed on the slides and sealed with nail polish. Once dried, the slides were visualized using an Axioskop 2 microscope (Zeiss) and Openlab 5.00 software (Improvision). The images were prepared using PhotoShop 6 (Adobe).

### Yeast two-hybrid analysis

*Saccharomyces cerevisiae* L40 strain was grown on YPD media [10 g of yeast extract (Melford), 20 g of bactopeptone (Melford) and 20 g of dextrose (Fisher) in 1 L at pH 5.8, supplemented with adenine at 0.01%; Sigma] at 30°C. If plates were required, agar was added per manufacturer's instructions (Melford). Minimal media (YC-W; Clontech) was used for the growth of cells containing pYesTrp2-Prey constructs, while selection with 300 µg ml^−1^ zeocin (Invitrogen) was used for cells containing pHybLex/Zeo-Bait constructs. β-Galactosidase was assayed by a kit by Pierce, following manufacturer's instructions. Briefly, two independent co-transformant clones were grown as liquid cultures in YC-W with 300 µg ml^−1^ zeocin at 30°C for 48 h. Cell density was quantified by measuring the optical density (OD) at 620 nm. Cell lysis solution and o-nitrophenyl-β-D-galactopyranoside (ONPG) was added to the cells. β-Galactosidase-mediated conversion of ONPG to o-nitrophenol was quantified by measuring absorbance at 420 nm using a 96-well plate reader (Wallace Envision, 2102 Multi-label reader). β-Galactosidase activity was calculated using the following equation: (1000 × Absorbance 420 nm) ÷ (t × V × OD 620 nm), where t is the assay reaction time in minutes and V is the volume of culture used in the assay in ml. β-Galactosidase activity of the cells was also assayed by a filter lift procedure (data not shown), and interactions assayed via histidine auxotrophy (data not shown); details of these approaches can be supplied.

### Purification of RAD51 and RAD51 paralogues, and antiserum generation

RAD51 and RAD51 paralogues were purified both as histidine (His)-tagged variants and as GST-tagged variants. The genes were C-terminally tagged with 10 × His by cloning the full-length ORFs into pET51b after PCR amplification; N-terminal GST fusions were generated by cloning in pGex-4T3 (Amersham). All proteins were expressed after transforming the plasmids into *E. coli* Rosetta 2 cells (Novagen). RAD51-His expression was induced with 1 mM IPTG for 2–4 h at 37°C, yielding soluble protein. All His-tagged RAD51 paralogues were expressed in the same way, but were purified from inclusion bodies. Purification was performed using a 5 ml Histrap column connected to an AKTAprime (both GE Healthcare). After induction, cells were harvested by centrifugation (5200 *g*, 20 min) and resuspend in 10–20 ml binding buffer (20 mM Na phosphate pH 7.5, 0.5 M NaCl, 20 mM imidazole, 1 mM DTT for soluble protein; supplemented with 8 M Urea for insoluble protein). For soluble protein, the cells were then lysed by sonication on an ice/water mix (10 cycles of 20 s on/30 s off, amplitude 18) and centrifuged (20 000 *g*, 4°C, 25 min). For insoluble protein, the cells were lysed using a dounce homogenizer and centrifuged at room temperature (20 000 *g*, 25 min). In either case, supernatant was then applied to the column after filtering (0.2 µm syringe filter for soluble, vacuum filter for insoluble). Proteins were recovered from the column in elution buffer (20 mM Na phosphate pH 7.5, 0.5 M NaCl, 400 mM imidazole, 1 mM DTT for soluble protein; supplemented with 8 M Urea for insoluble protein), after a wash in 30% elution buffer. Proteins were then dialysed (4°C, 3 times, using 30 volumes for 90 min per step) in non-denaturing buffer (for RAD51, RAD51-3, RAD51-4 and RAD51-6, 20 mM Tris-Cl pH 7.5, 0.5 M NaCl, 1 mM DTT, 50% glycerol; for RAD51-5, same buffer but with 0.2 M NaCl), centrifuged (20 000 *g*, 4°C, 15 min), snap-frozen on dry ice and stored at −80°C before being sent for antibody production. The conditions for expression and purification of the GST fusion proteins are described for GST pull downs (below). Proteins were purified from cell extracts using 1 ml GST trap HP columns connected to an AKTAprime (both GE Healthcare) following the manufacturers' instructions and using the binding and elution buffers described below. It was not possible to express GST–RAD51-5 solubly, and therefore untagged protein was purified from inclusion bodies using anion and cation exchange (data not shown). Polyclonal antiserum were raised (Scottish Blood Transfusion Board, Edinburgh) against the His-tagged proteins in lop-eared rabbits (RAD51 and RAD51-4), sheep (RAD51-3), rats (RAD51-6) or chicken (RAD51-5). For anti-RAD51-5 antiserum IgY was purified from the egg yolk using a chicken IgY purification kit (Pierce), according to manufacturer's instructions. Where necessary, the antisera were affinity-purified using GST fusion proteins. Here, the GST fusion proteins were eluted in 50 mM Na phosphate pH 8.0, 10 mM reduced glutathione, 200 mM NaCl, 1 mM DTT. Using a buffer exchange column (Bio-Rad) they were then moved to coupling buffer (4 M Guanidine HCl, 100 mM Na phosphate pH 6.4, 0.05% Na-azide), protein concentration determined (Bio-Rad microassay) and coupled to beads (Aminolink resin; Pierce) to form an affinity column. For affinity purification, the resin was washed with 5 ml 0.05% sodium azide/PBS (Sigma) and antiserum added. Approximately 100 µl of 0.05% sodium azide/PBS was added and allowed to flow through, after which the column was closed at the bottom, 500 µl of 0.05% sodium azide/PBS added, the top capped and incubated at room temperature for 1 h. Next, the column was washed with 12 ml of PBS and antiserum eluted (in 0.5 ml fractions) by addition of 4 ml of 100 mM glycine-HCl (pH 2.7). Fractions were neutralized by addition of 50 µl of 1 M Tris-HCl (pH 7.5) and protein concentrations determined (Bradford assay, Bio-Rad) after dilution with 40 volumes oH_2_O. The buffer of fractions to be used was exchanged into PBS using a Bio-Rad Econo-Pac 10DG column, following manufacturer's instructions.

### GST pull down

GST fusions were expressed from pGex-4T3 (Amersham) and untagged proteins were expressed from pRSF1b (Merck); in all cases full-length *T. brucei RAD51* or *RAD51* paralogue ORFs were cloned by PCR (primers available on request). Normally, Rosetta *E. coli* cells were transformed the GST fusion and untagged protein expression vectors to allow coexpression and GST pull down. In cases where coexpression was not seen, the GST fusion and untagged proteins were expressed individually and GST pull down performed by combining individual cells extracts. In either case, a 50 ml culture of *E. coli* cells carrying the appropriate overexpression vectors, maintained by selection with antibiotics, were grown at 20°C until an OD of ∼ 0.5, at which time protein expression was induced with 1 mM IPTG overnight. Cells were then harvested by centrifugation (3750 *g*, 20 min, 4°C), resuspended in wash/binding buffer (PBS, containing 1 mM DTT, 0.1% NP40, 0.1% DNaseI and protease inhibitors; Roche) and stored at −80°C. The resuspensions were allowed to thaw at room temperature and were then lysed by sonication (4 cycles: 10 s on, 20 s off) and centrifuged (20 000 *g*, 20 min, 4°C). Samples of the supernatant were retained as input. To perform the GST pull down, glutathione sepharose beads (100 µl per cell extract) were washed twice with 5 ml wash/binding buffer and then resuspended in 10 volumes of the same buffer, forming a bead-slurry. Supernatant from the *E. coli* extract, or extracts, was added to the slurry and incubated at 4°C with rotation for 1 h. The beads were then harvested by centrifugation (500 *g*, 2 min, 4°C) and washed four times with 5 ml of wash/binding buffer. After the final wash, 0.5 ml of elution buffer (50 mM Tris-Cl pH 8.0, 200 mM NaCl, 20 mM glutathione, 0.1% NP40, 1 mM DTT) was added to the beads and incubated at 4°C with rotation for up to 30 min. Proteins were eluted from the beads by centrifugation at 500 *g* for 2 min (4°C) and the supernatant (eluate) added to protein loading buffer. After running on an SDS-PAGE gel, proteins were visualized with unpurified anti-RAD51 paralogue antiserum (see above) or with anti-GST antiserum (Novagen).
