# Supplementary material for: Interactions among Trypanosoma brucei RAD51 paralogues in DNA repair and antigenic variation
Source: Mol Microbiol. 2011 May 25;81(2):434–56. doi: 10.1111/j.1365-2958.2011.07703.x (PMC3170485; doi:10.1111/j.1365-2958.2011.07703.x)
Supplement: Supplementary file 1 [file mmi0081-0434-SD1.pdf]

Interactions amongst *Trypanosoma brucei* RAD51 paralogues in DNA repair and antigenic variation

Rachel Dobson, Christopher Stockdale, Craig Lapsley, Jonathan Wilkes and Richard McCulloch*

University of Glasgow,
College of Medical Veterinary and Life Sciences,
Institute of Infection, Immunity and Inflammation,
The Wellcome Trust Centre for Molecular Parasitology,
Sir Graeme Davis Building,
120 University Place,
Glasgow,
G12 8TA,
U.K.

Phone: 44-141-330-5946
Fax: 44-141-330-5422

corresponding author (email: **richard.mcculloch@glasgow.ac.uk**)

Accession, or genome identification, numbers for the Rad51 paralogue-encoding genes used in this study. Systematic names (e.g., RAD51-3, RAD51-4 etc) are not intended to infer orthology, except between *T. brucei, T.cruzi and L. major*.

1. Excavata.

Trichomonas vaginalis	**(http://trichdb.org/trichdb/)**
RAD51	TVAG_204070
DMC1	TVAG_155030
RAD51-3	TVAG_426330
RAD51-4	TVAG_144570
Giardia intestinalis	**(http://giardiadb.org/giardiadb/)**
DMC1A	XP_001709425; GL50803_13104
DMC1B	XP_001710001; GL50803_13346
Trypanosoma brucei	**(http://tritrypdb.org/tritrypdb/)**
RAD51	Tb11.01.0360
DMC1	Tb09.211.1210
RAD51-3	Tb11.02.0150
RAD51-4	Tb11.02.4880
RAD51-5	Tb10.389.1770
RAD51-6	Tb927.3.5230
Leishmania major	**(http://tritrypdb.org/tritrypdb/)**
RAD51	AAC16334; LmjF28.0550
DMC1	LmjF35.4890
RAD51-3	LmjF33.2490
RAD51-4	LmjF11.0230
RAD51-6	LmjF29.0450
Trypanosoma cruzi	**(http://tritrypdb.org/tritrypdb/)**
RAD51	Tc00.1047053503801.30
DMC1	Tc00.1047053506885.310
RAD51-3	Tc00.1047053504153.220
TcrRAD51-4	Tc00.1047053503613.30
RAD51-5	Tc00.1047053510123.30
TcrRAD51-6	Tc00.1047053508075.20
Naegleria gruberi	**(http://genome.jgi-psf.org/Naegr1/Naegr1.home.html)**
DMC1	jgi|Naegr1|88262
RAD51	jgi|Naegr1|88260
RAD51-3	fgeneshNG_pg.scaffold_18000116
RAD51-4	jgi|Naegr1|45247
RAD51-5	jgi|Naegr1|88254

2. Chromalveolata

Thalassiosira pseudonona	**(http://genome.jgi-psf.org/Thaps3/Thaps3.home.html)**
RAD51	jgi|Thaps3|255596
RAD51-2	jgi|Thaps3|257784
RAD51-3	jgi|Thaps3|261577
Cryptosporidium parvum **(http://cryptodb.org/cryptodb/)**	
RAD51	cgd5_410
DMC1	cgd7_1690
RAD51-3	cgd2_4070
RAD51-4	cgd6_4800
Theileria annulata	**(http://old.genedb.org/genedb/annulata/)**
RAD51	TA07350
DMC1	TA07075
RAD51-3	TA12290

Plasmodium falciparum	**(http://plasmodb.org/plasmo/)**
RAD51	PF11_0087
DMC1	MAL8P1.76
RAD51-3	PFD0935c
Toxoplasma gondii	**(http://toxodb.org/toxo/)**
RAD51	59.m03654
DMC1	35.m00010
RAD51-3	645.m00312
Tetrahymnena thermophila	**(http://ciliate.org/index.php/home/welcome)**
RAD51	TTERM_00142330
DMC1	TTHERM_00459230
RAD51-3	TTHERM_01143840

3. Plantae

Arabidopsis thaliana	
RAD51	NP_568402
DMC1	JC4092
RAD51C	NP_566040
RAD51B	NP_180423
RAD51D	NP_172254
XRCC2	NP_201257
XRCC3	NP_851202
RECA1	Q39199.1
RECA2	NP_179539.2
RECA3	NP_187625.2

4. Amoebozoa

Entamoeba histolytica	**(http://www.sanger.ac.uk/cgi-bin/blast/submitblast/e_histolytica)**
RAD51	63.m00157; EHI_031220)
DMC1	10.m00382; EHI_050430)
RAD51-3	59.m00167; EHI_122860)
Dictyostelium discoideum	**(http://dictybase.org/)**
RAD51	DDB0168161; DDB0217219
RAD51-2	DDB0186043
RAD51-3	DDB018559
RAD51-4	DDB0186432
RAD51-5	DDB0188824
RAD51-6	DDB021753
RECA	DDB0169470

5. Opisthokonta

Encephalitozoon uniculi	**(http://microsporidiadb.org/micro/)**
RAD51	Q8SQX0_ENCCU
RAD51-2	Q8SW20_ENCCU
Schizosaccharomyces pombe	
RHP51	P36601
DMC1	O4263
RDH55	O14129
RDH57	Q9UUL2
Saccharomyces cerevisiae	
RAD51	NP_011021
DMC1	NP_011106
RAD57	NP_010287
RAD55	NP_010361
Ustilago maydis	
RAD51	AAC61878

REC2	A56244
Caenorhabditis elegans	
RAD51	O44246 O44246_CAEEL
Rfs-1	P34348 YK82_CAEEL
Drosophila melanogaster	
RAD51	BAA04580
SPND	NP_733200
SPNB	NP_476740
RAD51C	NP_610466
RAD51D	NP_573302
Homo sapiens	
RAD51	Q06609
DMC1	Q14565
XRCC2	O43543
XRCC3	O43542
RAD51B	O15315
RAD51D	AAC39719
RAD51C	AAC39604

Supplementary figure legends

Supplementary Figure 1. Generation of *RAD51-4* and *RAD51-6* mutants in bloodstream form *T. brucei*. A. For both genes, the RecA-fold core of the ORF (white) was deleted (Δ), removing the Walker A and B boxes (black) and replaced with cassettes encoding either blasticidin (*BSD*, see **B**) or puromycin (*PUR*) resistance. **B, C.** Southern blots of genomic DNA from wild type (WT) cells, two heterozygous mutants (+/- 1, 2) and two homozygous mutants (-/- 1, 2) probed with the ORFs of *RAD51-4* or *RAD51-6,* respectively. *RAD51-4* mutants' DNA was digested with *Age*I, and *RAD51-6* DNA by *Nde*I. Positions of size markers (in kb) are shown, and hybridising fragments from the WT alleles, or from *BSD*- or *PUR*-disrupted (::) alleles, are indicated. **D.** Reverse transcriptase PCR from WT, +/- , -/- or re-expresser (-/-/+) cells using primers specific for *RAD51-4* or *RAD51-6*; RNA Polymerase (Pol) I-specific primers demonstrate intact cDNA in the -/- mutants. RT+ denotes the inclusion of reverse transcriptase during cDNA synthesis, RT- denotes controls in which the enzyme was omitted.

Supplementary Figure 2. DNA damage sensitivity in *T. brucei* RAD51 and RAD51 paralogue mutants. A. The concentration of methyl methanesulphonate (MMS) that caused 50% growth inhibition (IC50) of wild type (WT) cells is compared with two (1, 2) independent heterozygous (+/-) and homozygous (-/-) mutants of *RAD51-4* and *RAD51-6*, as well as with -/- cells re-expressing the mutated protein (-/-/+) **B.** Comparison of the MMS IC50s of WT, *rad51*-/-, *rad51-3*-/-, *rad51-4*-/-, *rad51-5*-/- and *rad51-6*-/- cells. Values are averages from at least 3 experiments; bars indicate standard deviation.

Supplementary Figure 3. Clonal survival of *RAD51-4* (A) and *RAD51-6* (B) mutants *in vitro* when treated with phleomycin or methyl methanesulphonate (MMS). Cell survival is shown as a percentage of clonal growth when treated with the DNA damaging agent compared with growth in the absence of drug. For each graph, wildtype 3174.2 (wt), two independent heterozygous mutants (X+/-, Y+/-), and two independent homozygous mutants (X-/-, Y-/-) are shown at the drug concentrations indicated; in addition, RAD51 paralogue re-expresser cells (-/-/+) are shown for the MMS assay. The graph shows the means of triplicate data, with 95 % confidence intervals indicated by error bars; growth was measured 10-14 days after the plating the cells out at 1 cell/well in 96 well plates.

Supplementary Figure 4. RAD51 subnuclear foci in *T. brucei* bloodstream stage cells. Example of a wild type (WT) *T. brucei* cells with differing numbers of discernible sub-nuclear RAD51 foci following 18 hrs growth in 1.0 $\mu g.ml^{-1}$ phleomycin are shown. For comparison, the lack of detectable RAD51 in *rad51*-/- cells is shown (top). Cells were visualised by differential interference contrast (DIC; right centre), the DNA was stained with DAPI (left centre), and RAD51 (left) was visualised by indirect immunofluorescence using a polyclonal anti-RAD51 antiserum and SFX-conjugated goat-derived anti-rabbit secondary; merged DAPI and RAD51 images are shown (centre), as well as merged DAPI, RAD51 and DIC images (right).

Supplementary Figure 5. RAD51 localisation in bloodstream form *T. brucei rad51-4*-/- and *rad51-6*-/- cells. Examples are shown of RAD51 localisation in *rad51-4*-/- and *rad51-6*-/- cells in the absence of induced DNA damage, or following 18 hrs growth in 1.0 $\mu g.ml^{-1}$ phleomycin [BLE]. The organisation of the images, and the way RAD51, DNA and cells were visualised, is as described in Supp Fig. 3.

Supplementary Figure 6. RAD51 localisation in bloodstream form *T. brucei rad51*-/-, *rad51-3*-/- and *rad51-5*-/- cells. Examples are shown of RAD51 localisation in *rad51*-/-, *rad51-3*-/- and *rad51-3*-

/- cells following 18 hrs growth in 1.0 μg.ml^{-1} phleomycin [BLE]. The organisation of the images, and the way RAD51, DNA and cells were visualised, is as described in Supp Fig. 3.

Supplementary Figure 7. RAD51 expression in *T. brucei* RAD51 paralogue mutants. Western blot of whole cell extracts, after SDS-PAGE, from wild type (wt), *rad51*-/-, *rad51-3*-/-, *rad51-4*-/-, *rad51-5*-/- and *rad51-6*-/- cells, probed with anti-RAD51 antiserum. Expression of RAD51 is indicated, as well as a cross-reacting band in all smaples (*), and a smaller band seen only in the RAD51 paralogue mutants (?). Size markers are shown.

Supplementary Figure 8. Analysis of the switching mechanisms of *RAD51-4 and RAD51-6* mutant cell lines. The graph shows the VSG switching mechanism of cell lines as a percentage of the total analysed. Wildtype strain 3174.2 (wt), two independent heterozygous mutants (X+/-, Y+/-), two independent homozygous mutants (X-/-, Y-/-) and RAD51 paralogue re-expresser (X-/-/+) cells are shown. The assay allowed differentiation of *in situ* transcriptional switching (*in situ*), expression site gene conversion (ES GC) and *VSG* gene conversion (VSG GC). Switch reactions that could not be assigned in these categories are indicated as “unknown”. The graph shows the means of triplicate data, with the 95 % confidence intervals indicated by the error bars.

Supplementary Figure 9. Western analysis showing the specificity of anti-RAD51 paralogue antibodies. For each protein western blots are show of three SDS-PAGE gels: GST-RAD51 (51) and GST-RAD51 paralogue (numbered 3, 4, 5, 6, corresponding with RAD51-3, RAD51-4, RAD51-5 and RAD51-6, respectively) recombinant proteins purified from *E. coli* (left blot); whole cell extracts from wildtype (wt) and *rad51* paralogue homozygous mutant (-/-) *T. brucei* cells probed with un-purified antiserum (middle blot) and affinity purified antiserum (right blot). Arrows indicate the predicted size of the native or GST-tagged RAD51 paralogue proteins; size markers are indicated (kDa).

Supplementary Figure 10. Synteny between *T. brucei* and *L. major* in the genomic environment around *T. brucei RAD51-5*. Percent sequence identity (large font) and similarity (small font) are shown when the predicted amino acid sequences of the genes surrounding *T. brucei RAD51-5* were compared with orthologues on *L. major* chromosome 33; ORFs are indicated as red or blue arrows; *T. brucei RAD51-5* is indicated by a dark blue arrow, and a potential *RAD51-5*-like gene in *L. major* by a light blue arrow.

Supplementary Figure 11. An alignment of *T. brucei* and *T. cruzi* RAD51-5 with the predicted amino acid sequence of a putative RAD51-5 orthologue in *L. major, L. braziliensis,* and *L. infantum*. The alignment was performed using Clustal X; residues that are identical in ≥ 30 % of the sequences are shown in black; those that are conserved in ≥ 30 % are in grey. The positions of conserved RecA/Rad51 features in TbRAD51-5 and TcRAD51-5 are indicated: Walker A and B motifs, including critical residues (circles), are indicated in green, and RecA homology upstream of the Walker A motif is indicated by a dashed line.

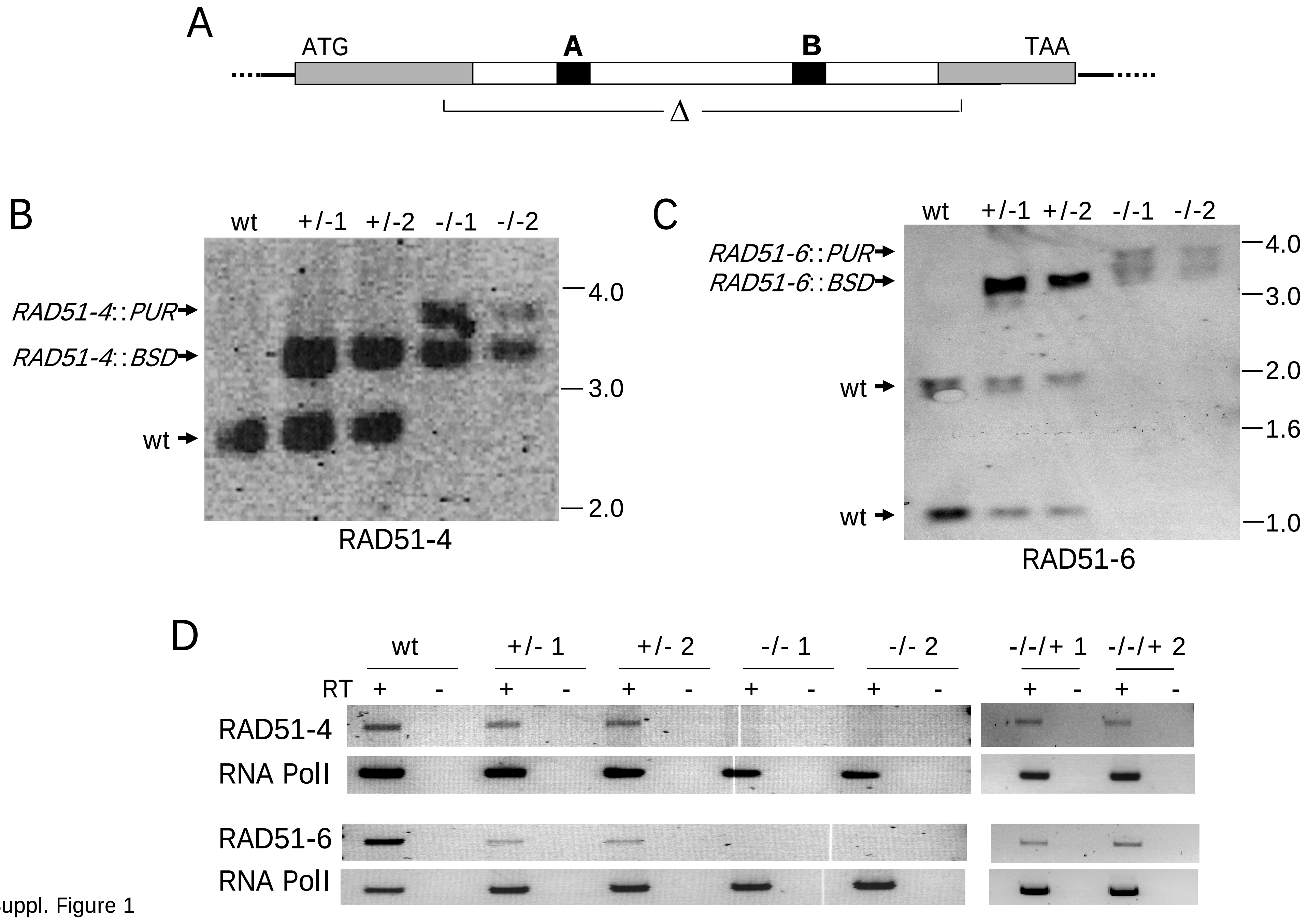

Suppl. Figure 1

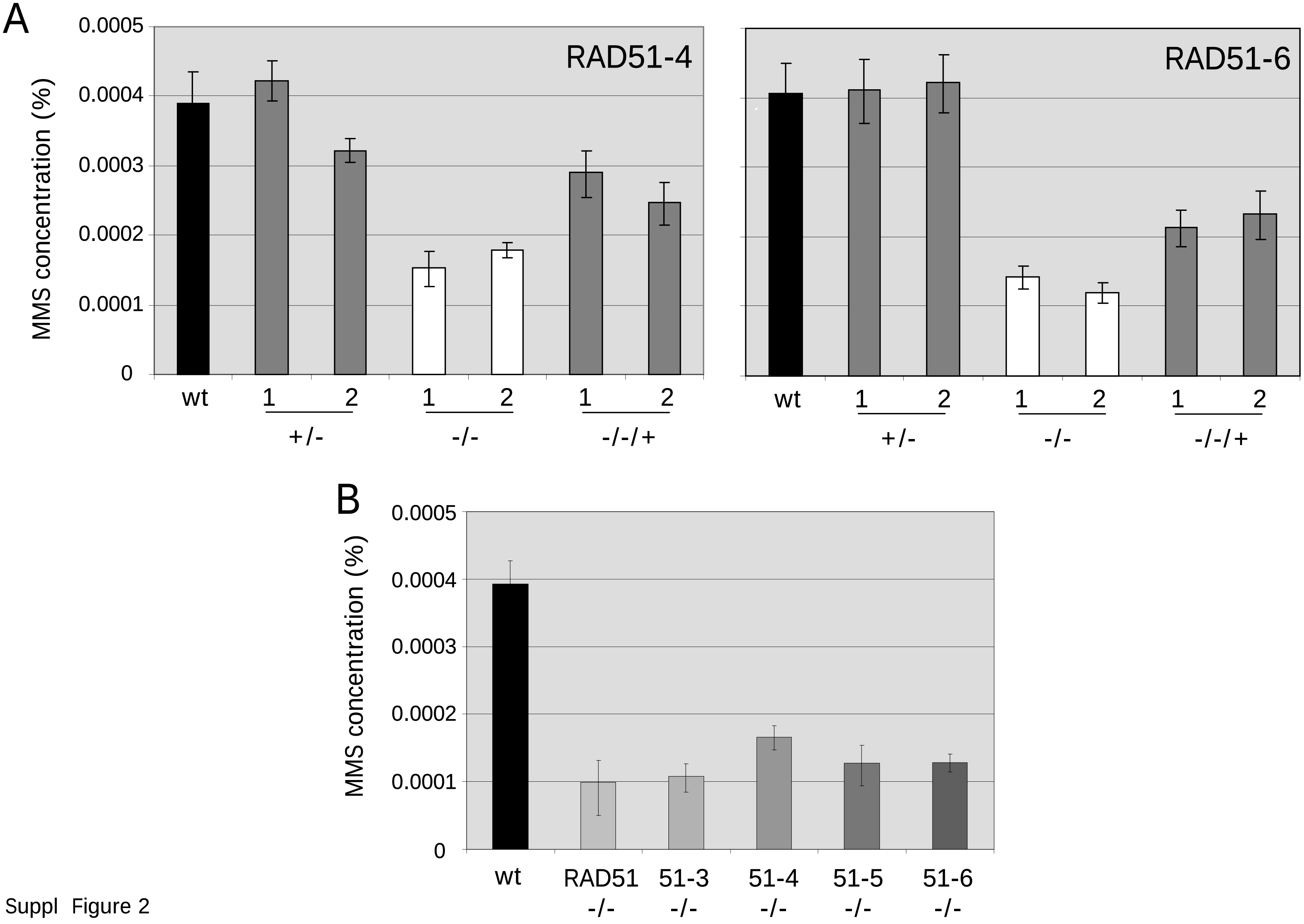

Suppl Figure 2

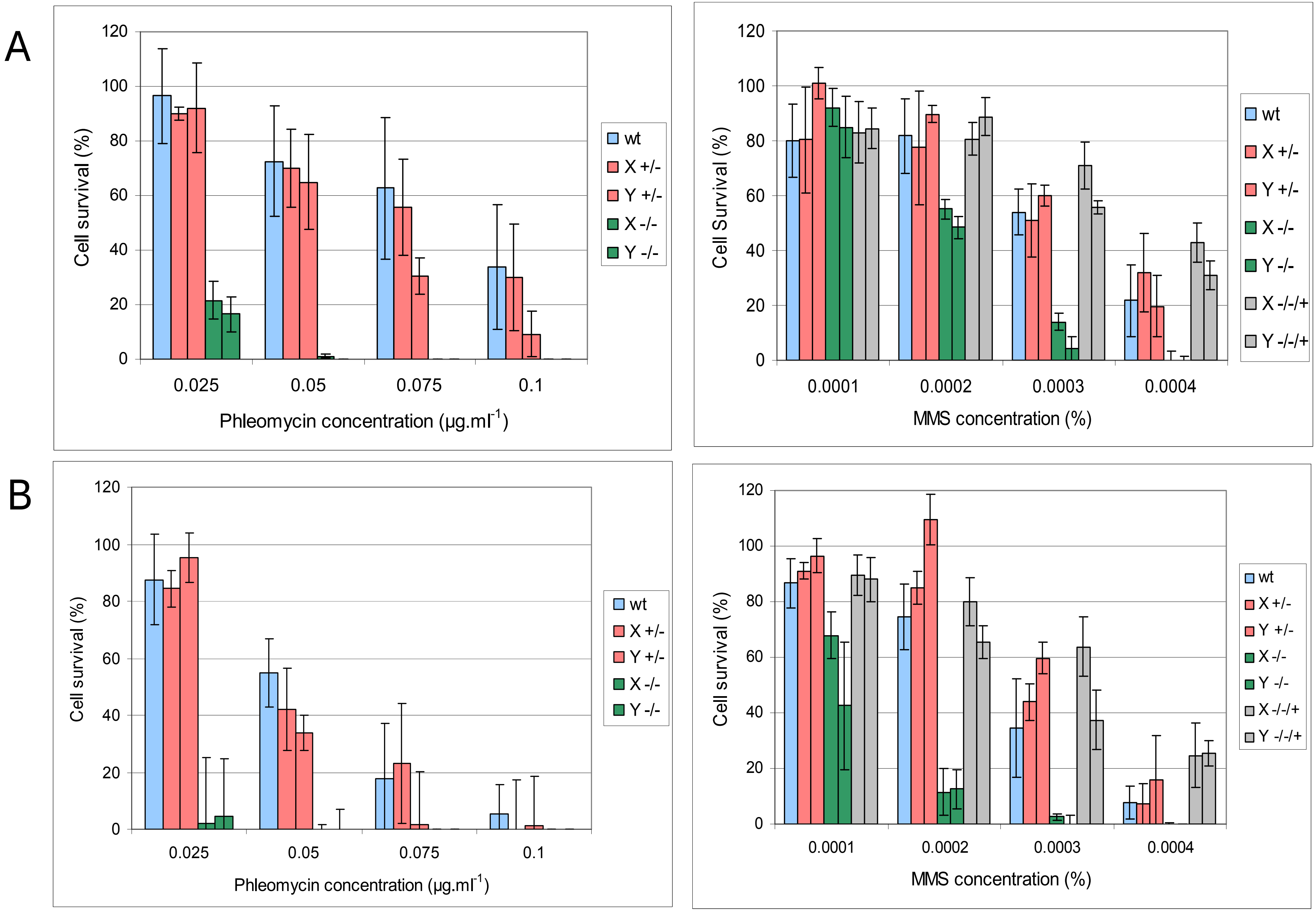

Suppl Figure 3

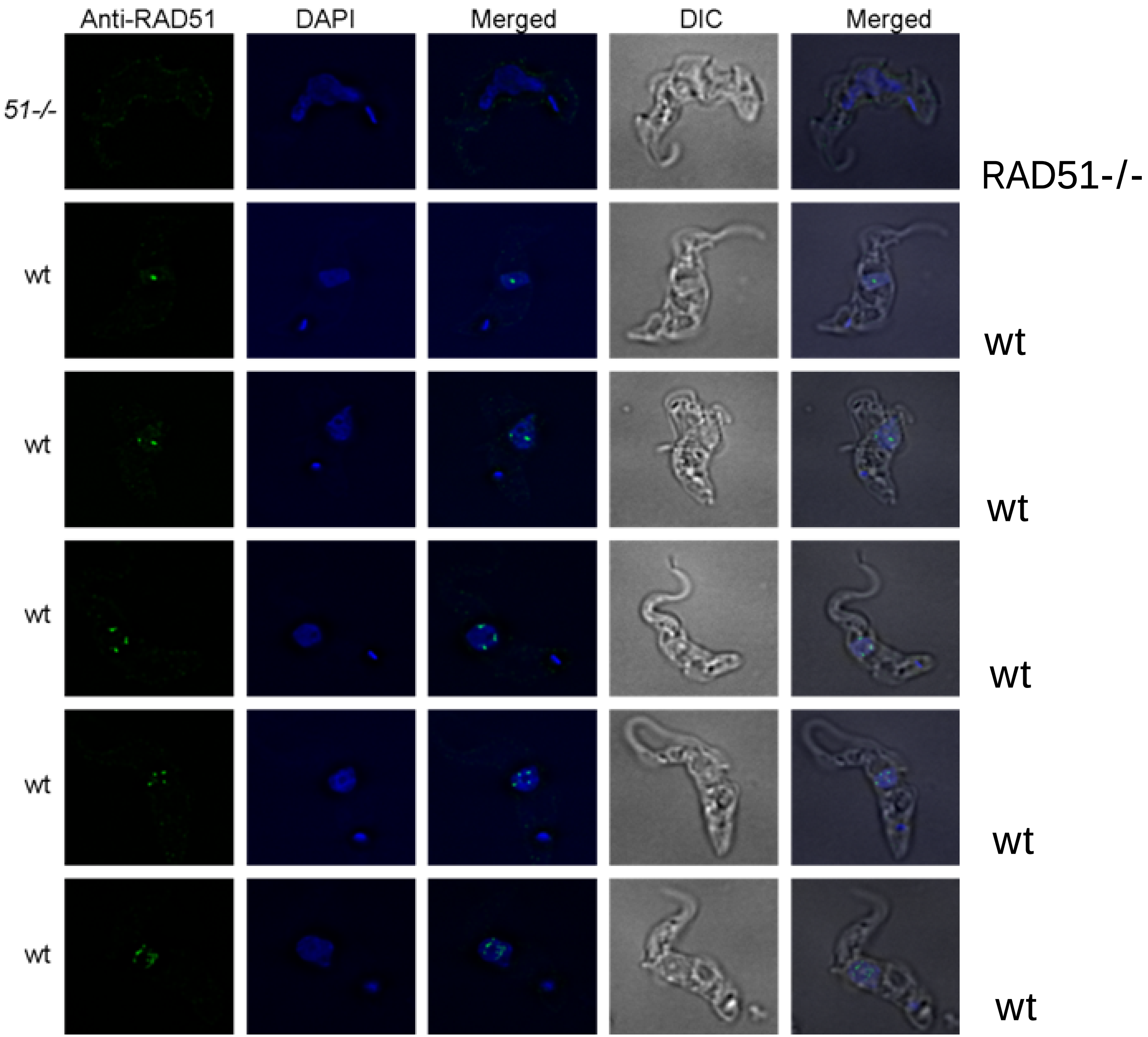

Suppl Figure 4

18 hrs, 1.0 ug.ml^{-1} phleomycin

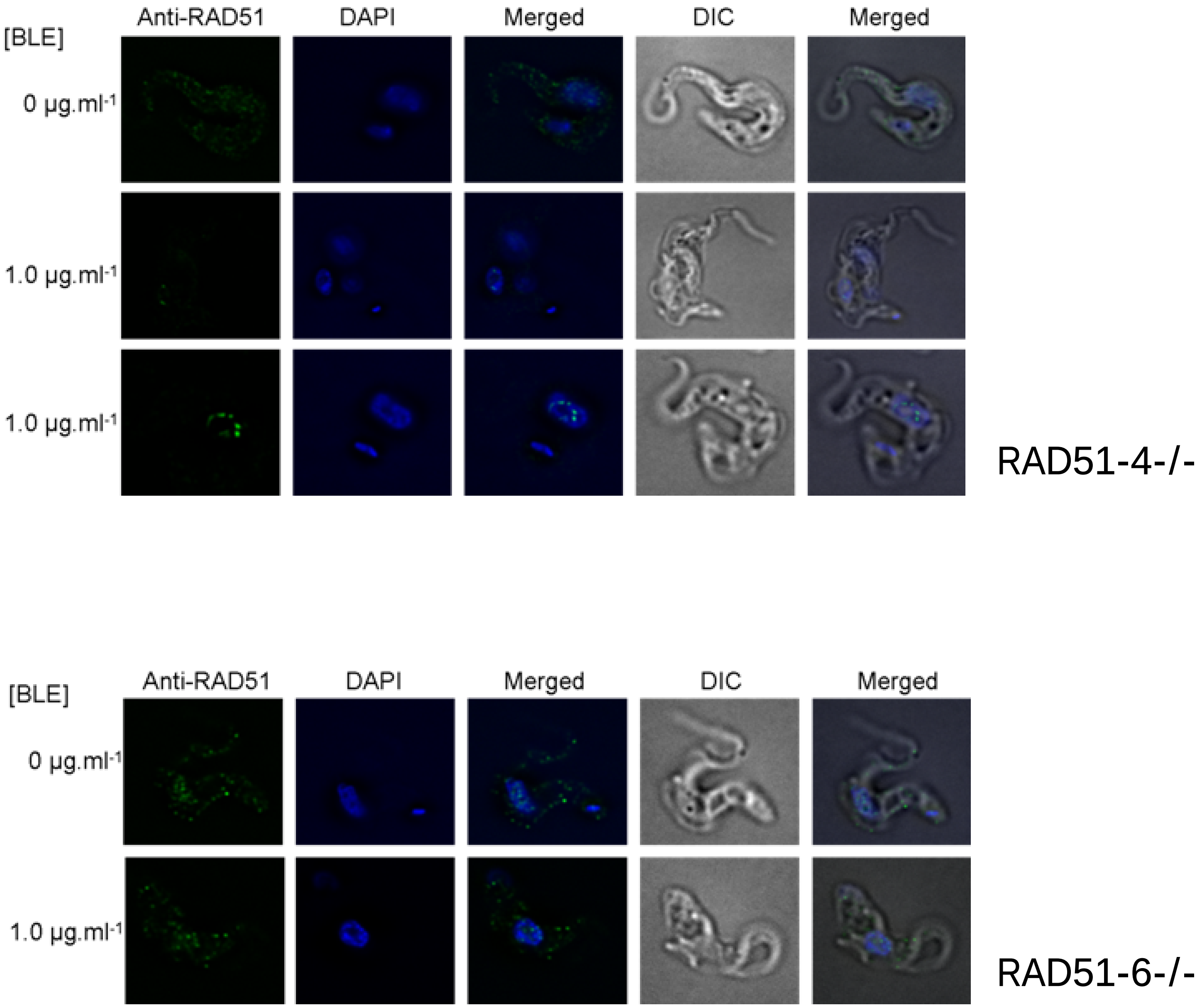

Suppl Figure 5

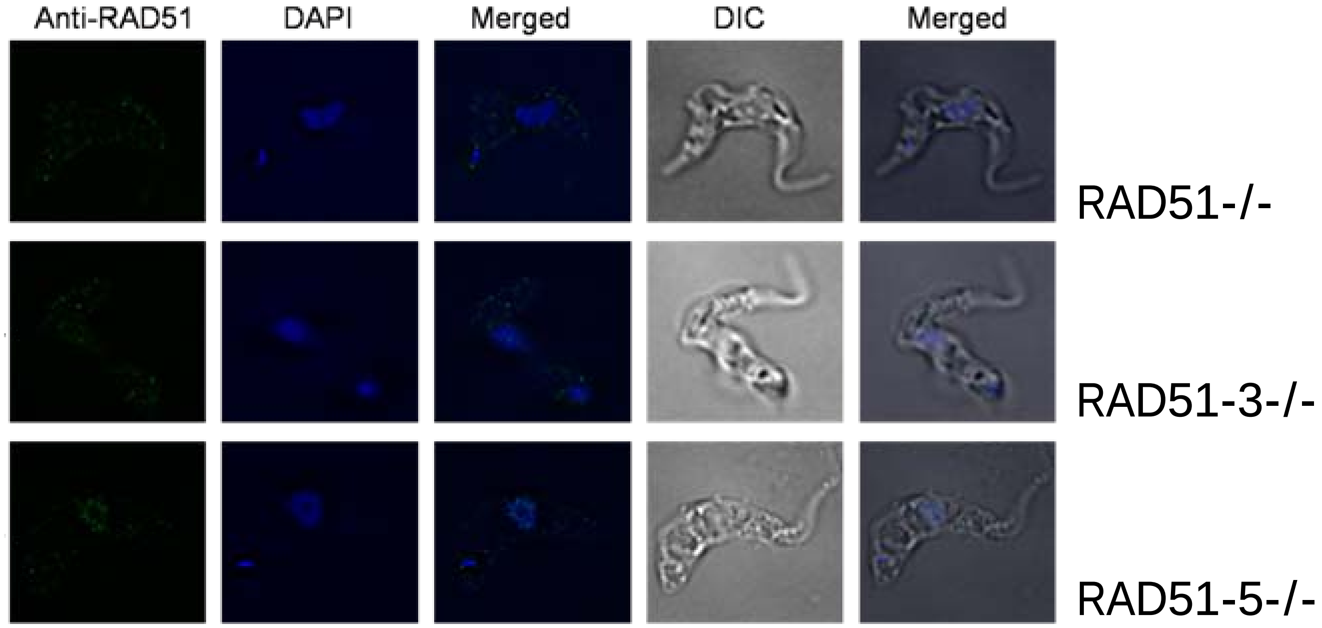

18 hrs, 1.0 ug.ml^{-1} phleomycin

Suppl Figure 6

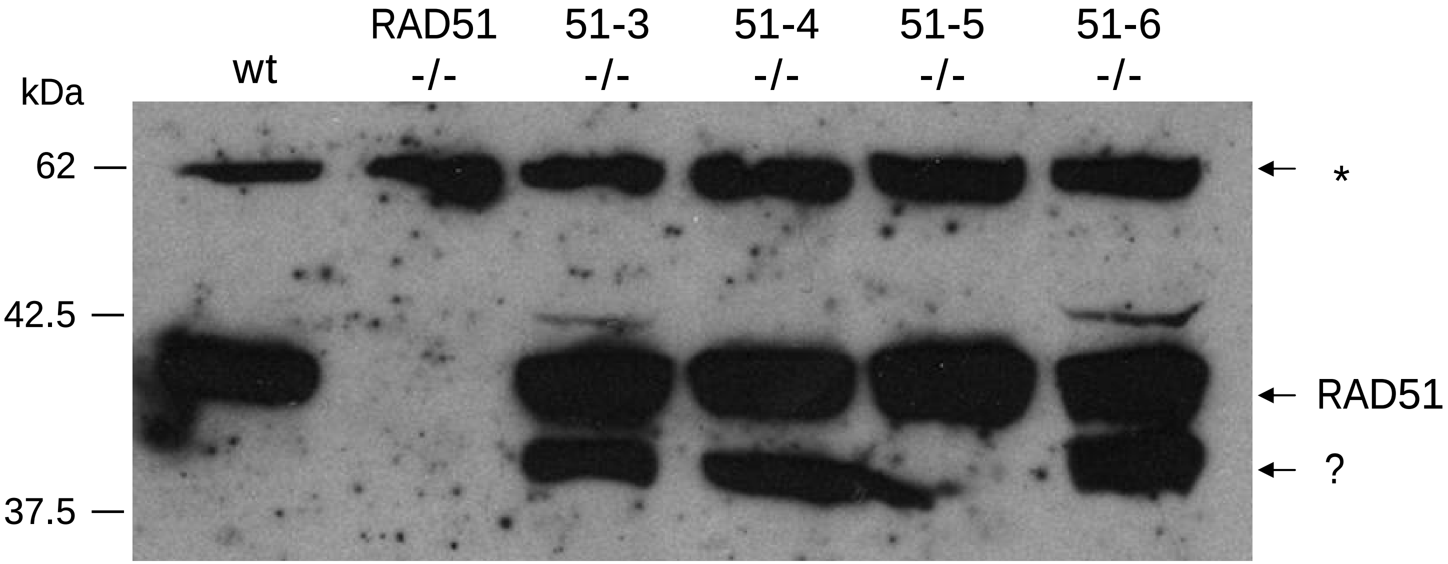

Suppl Figure 7

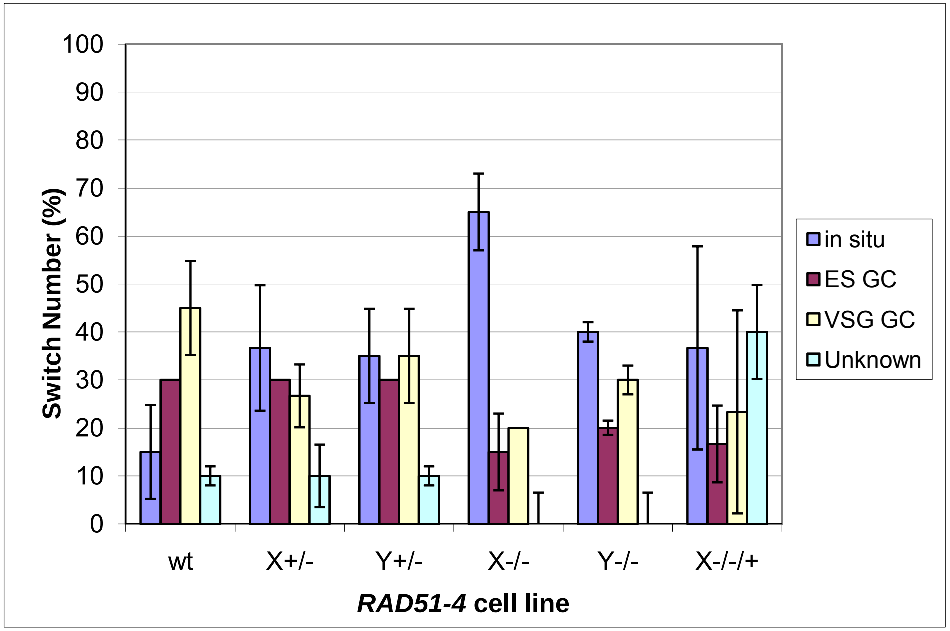

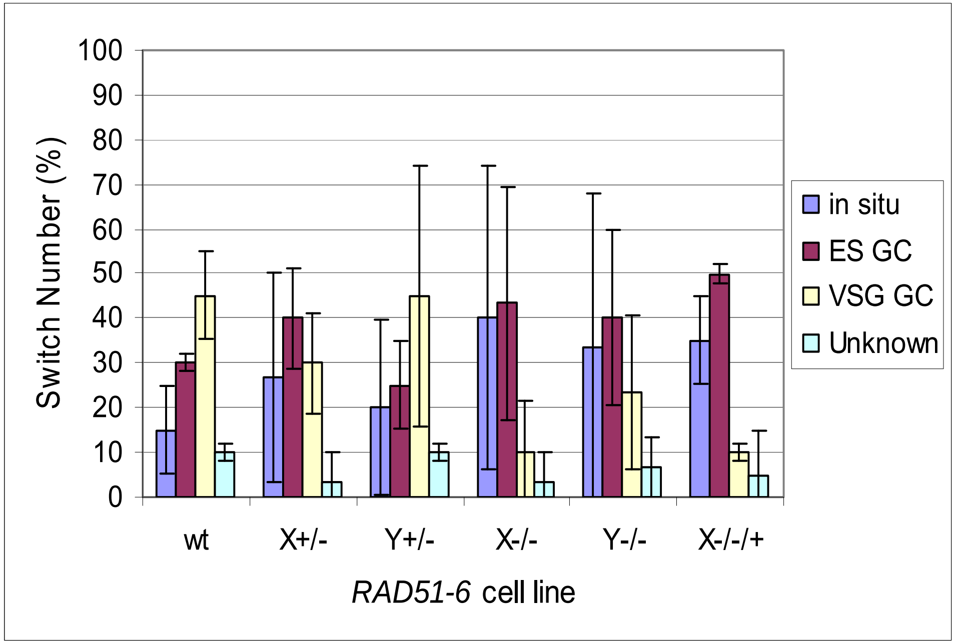

Suppl Figure 8

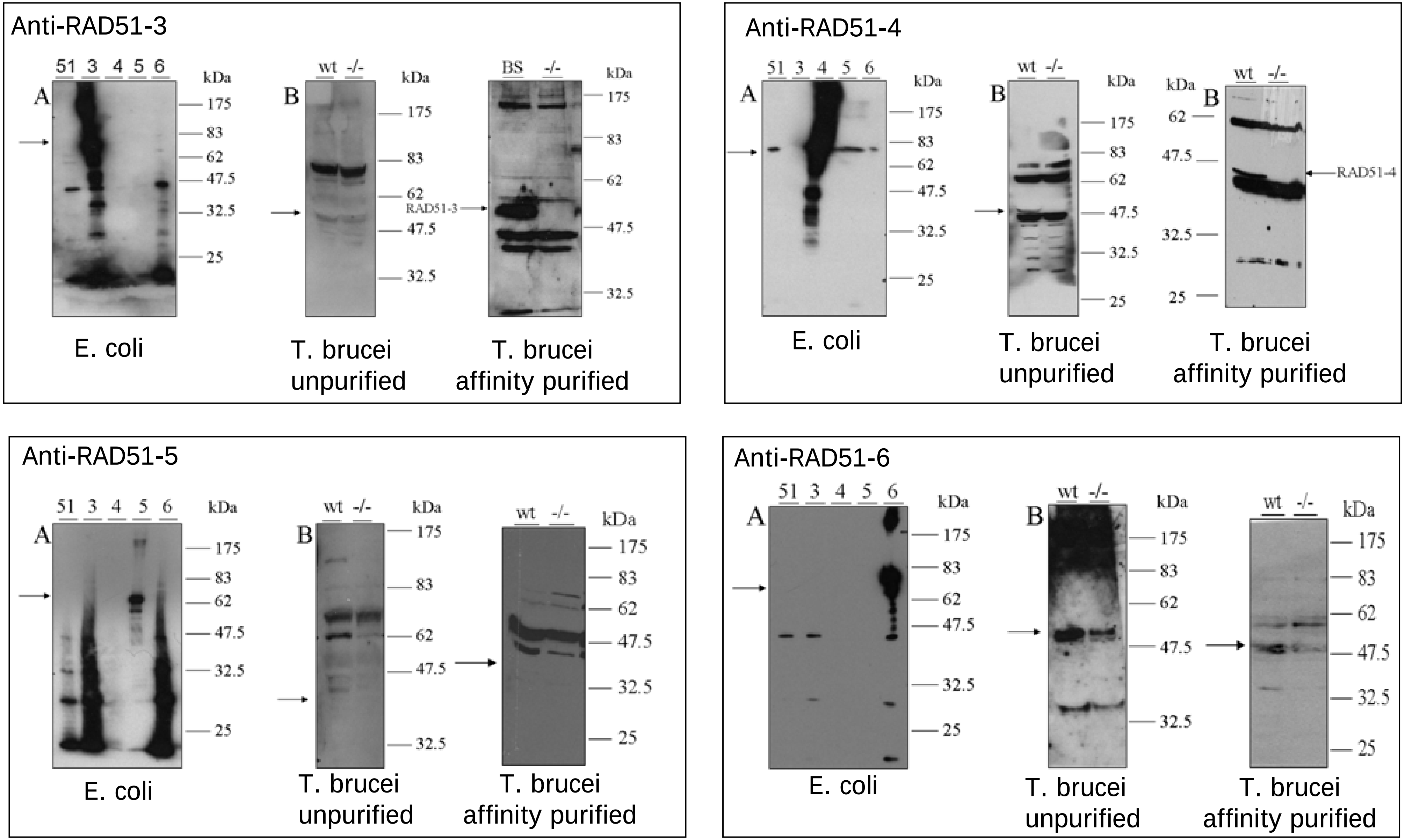

Suppl Figure 9

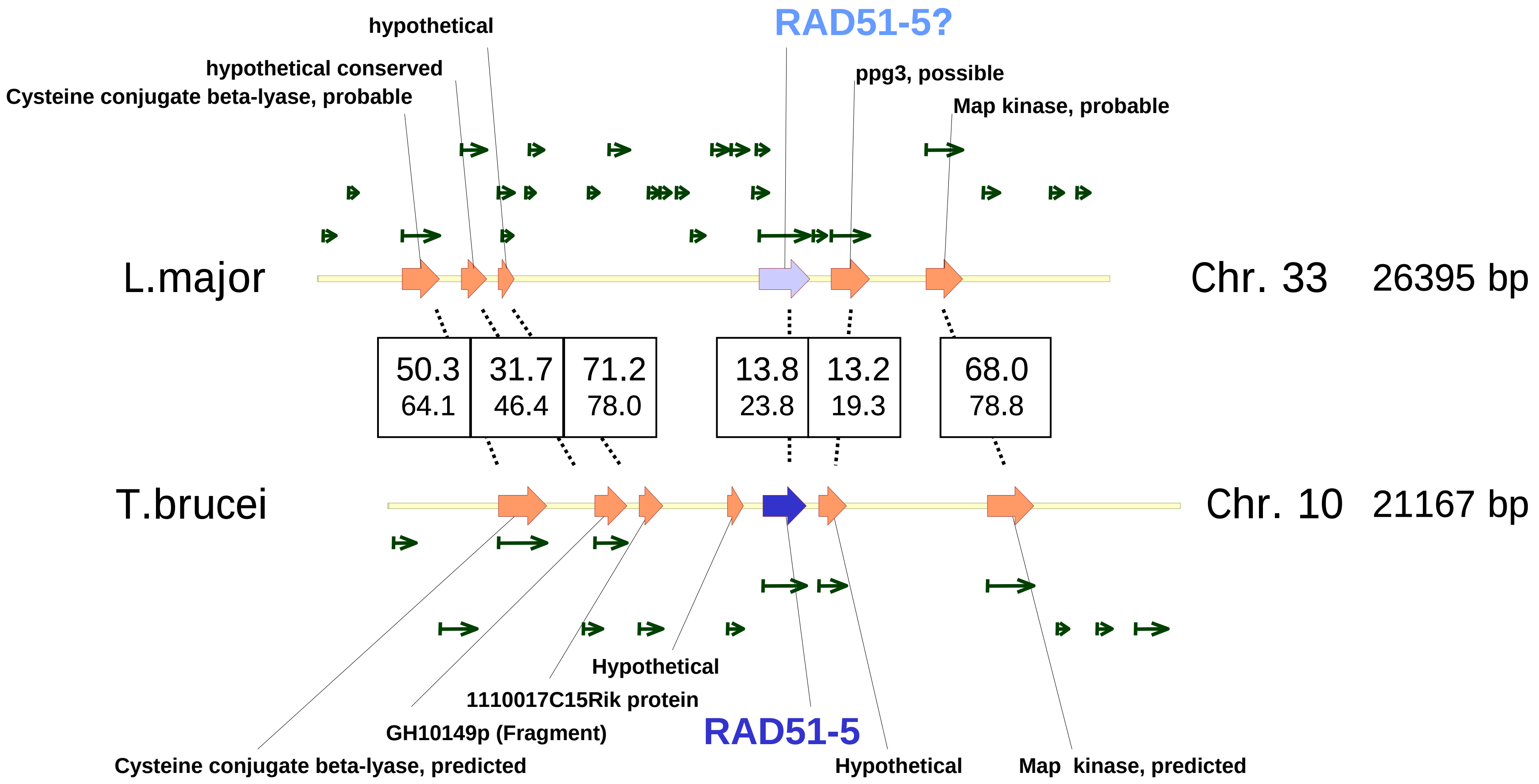

Suppl Figure 10

```
LmaRAD51-5?    1 ------------------------------------------------------------
LifRAD51-5?    1 ------------------------------------------------------------
LbrRAD51-5?    1 MQSTTASWETMSDSAARARVLRLRQSATPLQDVYGAFSPWQHLSTSLPGLDSLLSVAGVC
TbrRAD51-5     1 ------------------------------------------------------------
TcrRAD51-5     1 ------------------------------------------------------------

LmaRAD51-5?    1 -------------------------------------------------MTMYRQWYHDE
LifRAD51-5?    1 -------------------------------------------------MTMCRQWYHDE
LbrRAD51-5?   61 EGVSGSDDRSGVESTIGGLPVGGLHELYGPPLSGKSWLLRRVGAAYVRRMTAYRQWYHDE
TbrRAD51-5     1 ------------------------------------------------MSVCPPPWTGMT
TcrRAD51-5     1 ------------------------------------------------MAVMPPPWEALT

LmaRAD51-5?   12 LERVSKWAVETPRSTRTYDDEAQANKDDEGLCGEGEVADTWADSAGASPVTAMEEWDLYV
LifRAD51-5?   12 LELASKWAVETPRSTHTYDDEAHAKKDDEGLCSEGEVADTWADSTGASPVTAMEEWDLYV
LbrRAD51-5?  121 LERVSKRAVEATLFTRKTDNEARAAKDDEDVDSEAEDAHICANSANPSPITAMEEWDLYV
TbrRAD51-5    13 AEEISQRLALLTSQTILQHDLNRRFTQHAVFSTGSEELDRLLPDGGMTCGTVLEVFG---
TcrRAD51-5    13 AEDVSRRLAALAASASSVLEVGRCFSREAVFSTGSAAIDRLLPDGGVACGTVLEIFG---
                              A
LmaRAD51-5?   72 CLVSGAGAGSATHQLTAPPPSPSLLSPDVRSWVEELVAPFDSSSTLDRQGEAFVTGPAHW
LifRAD51-5?   72 CLVSGAGASSTTHQLTAAPPSPSLLSPDVRSWVEELVAPFDSSSTLDRQGEAFVTGPAHY
LbrRAD51-5?  181 CLVRGAGADSKTHGLTASPPSPSLLSPDVRSWLKELVAPFDSSSTLDRQVESIVTGPVHG
TbrRAD51-5    70 ------------------PP-----SGGKSRLVRRMISSFAAQGALEWTTRG-VDVGICD
TcrRAD51-5    70 ------------------PP-----AAGKSHLVQQMVAAFAARGSVQWTAAKRSRTAVGG

LmaRAD51-5?  132 HASTHRRQQQRDYAEQHIHFRVVHSPNELLAFLEHLGSNAALAPSAGAAFADAAPSIVPP
LifRAD51-5?  132 HASEHRRQQQRDYAEQHIHFCVVRSPNELLAFLEHLSGDAALAPSAGTTFADAAPSIVPP
LbrRAD51-5?  241 YSSSH-QQQHRDYAEQHIHFRVVHSPNELLAFLECLGGGAASVPSTSTVFTNTTYSAAPP
TbrRAD51-5   106 GSPSKGLEVQQECLRARAEEC-CRPRE--WCVFYVLSDPSSLNPRHLREELEKALQRAML
TcrRAD51-5   107 NSVDRQQQQQQQQPHEEVEESKCGPCD--WSVFLLVSDPSSVSPQHIHELLKKALASATA

LmaRAD51-5?  192 VPLVASQQQPSSLQLSRSSSTTTARGQKRRRSPRTAVSSSSSVSPSCGKLPQRTWRLQRQ
LifRAD51-5?  192 APLVASQQQPSSLQLSCGSSATTARGQKRRRSPRTAVSSSSSVSPFRGNFPQRTWRLQRQ
LbrRAD51-5?  300 VPRVALQQQLLPSQLARGSLTTAMRGQKQHRSPGTTMPSSPSFPLACGSLPQCTWRLQRQ
TbrRAD51-5   163 CDVHCEG------EIERLLDDIIGRVQVVNFATLNDLLNFFRFLYHEEYPSSMHHANQRR
TcrRAD51-5   165 NSVQCDG------DADSLPSVMLKKIKFVPFKSPNDLLVFFRFLSRVEYFESCHHANRRR
                    B
LmaRAD51-5?  252 RLLLLDGLDALWLHPSLGNHCATHTGQWFAEELHRRLRAVLLPRLSYAASNSAVVTPSYA
LifRAD51-5?  252 RLLLLDGLDALWLHPSLGNHCATHTGQWFAEELHRRLRAVVLPRLSYAASNSAVLTPSYA
LbrRAD51-5?  360 RLLLLDGLDALWLHPSLGNHSATHTGQWFAEELHRQLRTVLLPRLGYAASDSTLLTAPSS
TbrRAD51-5   217 ALVVIDSVARLWDHPTCG--ATKHARHWAAAELVRELRNVIMLGNGWRGEYCGNIDSHHL
TcrRAD51-5   219 VLVVVDNVARLWEHPTCG--ATNHARHWMAAALVREVRNAILIGNGWRDVGSGEVEKRER

LmaRAD51-5?  312 TAAASPTSPYPHHTLYSTVVFTNGCNGSSRGILNPQQLEARLAGPVGGAEGWTATLPRPS
LifRAD51-5?  312 TAAASPTSSHPYHTLYSTVVFTNGCNGSSRGILNPQQLEARLAGPVGGAKGWMATLPRPS
LbrRAD51-5?  420 TAAAAPMSPHPQHVLYSTVVFTNGCHGSSRGFLTAQQLEARLAGPAGGVEGWAATLPRPS
TbrRAD51-5   275 DTEAGNVFCS-----VQAANAGTSVGGSVAVVLVNGCTNTRYCHTVVG------PLGVP-
TcrRAD51-5   277 HHHAHDASVS-----GQEISV-DCRGGSVAVVFVNGCTSVYHRKLATERTLPSKPLGVP-

LmaRAD51-5?  372 GNAVWCRAADTRCLVEPAHPGLVSLPTPSSSYVHPARVSHGPGAMRQSCRGMTKAVGPSN
LifRAD51-5?  372 GNAVWCRAADTRCLVEPAHPGLVSLPTPSSSYVCSARVSHGPGAMRQSSRGMAKTFGPSN
LbrRAD51-5?  480 GNAAWCRAVDTRCLIEPAHLGLVSILTPSFSYVRPAMAPHESSAVYQNGRRITGTFRQST
TbrRAD51-5   323 --LWLAAAADIRLFIEPTST--------SGDYPPTTGEDDDGMLCTHQYGAAKHVLA---
TcrRAD51-5   330 --VWLAAAADVRLFVEPLCN--------ADWFLQHDRSSGDVCGTQQSEHAARATMR---

LmaRAD51-5?  432 ANSRESLVTVVKGGSCVAATWVLRDVTGGEQET
LifRAD51-5?  432 TNSLESLVTVVKGGSRVAATWLLRDVAGGAQES
LbrRAD51-5?  540 ANYLESLVTVVKGGSRVAATWVLRNAADGEQEA
TbrRAD51-5   370 -------VRVAKGSGSSVPRVGNIFLP------
TcrRAD51-5   377 -------VRLVKGGRTVGGEVTAV---------
```

Suppl Figure 11
